# Single‐cell transcriptomic analysis of the senescent microenvironment in bone metastasis

**DOI:** 10.1111/cpr.13743

**Published:** 2024-09-04

**Authors:** Shenglin Wang, Lu Ao, Huangfeng Lin, Hongxiang Wei, Zhaoyang Wu, Shuting Lu, Fude Liang, Rongkai Shen, Huarong Zhang, Tongjie Miao, Xiaopei Shen, Jianhua Lin, Guangxian Zhong

**Affiliations:** ^1^ Department of Orthopaedics Fujian Institute of Orthopaedics, The First Affiliated Hospital of Fujian Medical University Fuzhou China; ^2^ Department of Bioinformatics, Fujian Key Laboratory of Medical Bioinformatics School of Medical Technology and Engineering, Fujian Medical University Fuzhou China; ^3^ Institute of Precision Medicine Fujian Medical University Fuzhou China; ^4^ Key Laboratory of Gastrointestinal Cancer (Fujian Medical University) Ministry of Education Fuzhou China

## Abstract

Bone metastasis (BM) is a mortality‐related event of late‐stage cancer, with non‐small cell lung cancer (NSCLC) being a common origin for BM. However, the detailed molecular profiling of the metastatic bone ecosystem is not fully understood, hindering the development of effective therapies for advanced patients. In this study, we examined the cellular heterogeneity between primary tumours and BM from tissues and peripheral blood by single‐cell transcriptomic analysis, which was verified using multiplex immunofluorescence staining and public datasets. Our results demonstrate a senescent microenvironment in BM tissues of NSCLC. BM has a significantly higher infiltration of malignant cells with senescent characteristics relative to primary tumours, accompanied by aggravated metastatic properties. The endothelial‐mesenchymal transition involved with SOX18 activation is related to the cellular senescence of vascular endothelial cells from BM. CD4Tstr cells, with pronounced stress and senescence states, are preferentially infiltrated in BM, indicating stress‐related dysfunction contributing to the immunocompromised environment during tumour metastasis to bone. Moreover, we identify the SPP1 pathway‐induced cellular crosstalk among T cells, vascular ECs and malignant cells in BM, which activates SOX18 and deteriorates patient survival. Our findings highlight the roles of cellular senescence in modulating the microenvironment of BM and implicate anti‐senescence therapy for advanced NSCLC patients.

## Graphical abstract

The NSCLC bone metastases demonstrate a senescent microenvironment characterized by aggravated metastatic properties of cancer cells, vascular ECs undergoing EndMT and increased infiltration of CD4Tstr cells. The SPP1 pathway may induce cellular communication among T cells, vascular ECs and malignant cells, shaping a pro‐angiogenic microenvironment that contributes to bone metastasis.

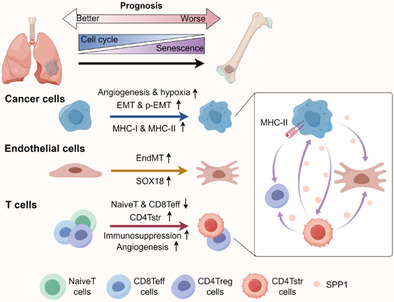

## 1 INTRODUCTION

Lung cancer causes nearly 1.8 million deaths worldwide annually,^1^ with non‐small cell lung cancer (NSCLC) accounting for over 80% of these cases.^2^ Bone metastasis, an undesirable incident resulting in dramatically increased mortality in cancer patients, occurs frequently in approximately 30%–40% of NSCLC patients.^3^ Patients afflicted with bone metastases (BM), even receiving symptomatic treatment combined with surgery, suffer an average survival time of less than 1 year and a 5‐year survival rate of below 5%.^4^ Therefore, an improved understanding of the mechanisms underlying bone metastasis is of significance to prolong the prognosis of advanced lung cancer patients.

The tumour microenvironment (TME) has been given an increasing spotlight due to its supporting role in tumour initiation, development, metastasis and drug resistance.^5^ Compared to other metastatic organs, the bone marrow microenvironment presents distinct features that promote the metastatic outgrowth of lung cancer cells, including the unbalanced activation of osteoclasts and osteoblasts and the caused release of embedded growth factors. This vicious cycle of bone destruction favours the seeding and proliferation of disseminated malignant cells around metastatic foci.^6^ Apart from bone resorption, the bone marrow is also characterized by an immunocompromised microenvironment. The presence of abundant immature and inhibitory immune cells in the premetastatic niche leads to the failure to kill disseminated cancer cells.^7^ Moreover, the interplay of cancer‐associated fibroblasts with stromal and immunosuppressive cells also contributes to bone metastasis.^8^ However, several trials have reported inconsistent results of the combined therapy regimen of immunotherapy with chemotherapy or bone‐targeted therapy in bone‐metastasis patients with advanced lung cancer.^9^, ^10^, ^11^ Detailed molecular profiling of various cell types in the TME is therefore required to uncover the metastatic mechanisms involved in advanced lung cancer.

Single‐cell RNA sequencing (scRNA‐seq) technology provides insights into the gene expression profiles of primary NSCLC at single‐cell resolution, revealing extensive heterogeneity of immune and stromal landscapes.^12^, ^13^ There are also several studies engaging in the metastatic progression of lung cancer. According to Kim's study, lung adenocarcinoma progressing from early stage to lymph node and brain metastases demonstrates a pro‐tumoral and immunosuppressive milieu with increasing infiltration of monocyte‐derived macrophages and dendritic cells, and with progressive T‐cell exhaustion.^14^ Targeted therapies induce decreased macrophages and activated cytotoxic T cells in residual disease, whereas they shape an immunosuppressive phenotype in progressive disease, as observed in primary lung biopsies and metastases including lymph nodes, pleura, liver, or brain.^15^ A recent study by Wang et al. identified a subpopulation of malignant cells responsible for driving brain metastasis in lung cancer.^16^ Until now, a comprehensive depiction of the metastatic bone ecosystem of NSCLC at the single‐cell resolution remains elusive.

In this study, we utilized scRNA‐seq to delineate the metastatic bone landscape of NSCLC by comparing the transcriptomic features of BM with primary tumours (PT). We identified a senescent microenvironment in the metastatic bone niche of NSCLC characterized by aggravated metastatic properties of cancer cells, vascular ECs undergoing endothelial‐mesenchymal transition (EndMT), and increased infiltration of CD4Tstr cells. Additionally, the SPP1 pathway may induce cellular communication among T cells, vascular ECs, and malignant cells, shaping a pro‐angiogenic microenvironment that contributes to bone metastasis. Our findings provide insights into the comprehensive understanding of molecular characteristics involved in NSCLC bone metastasis and aid the development of targeted therapies for metastatic patients.

## 2 RESULTS

### 2.1 Landscapes of cell composition in primary and bone‐metastatic microenvironment

**FIGURE 1:**
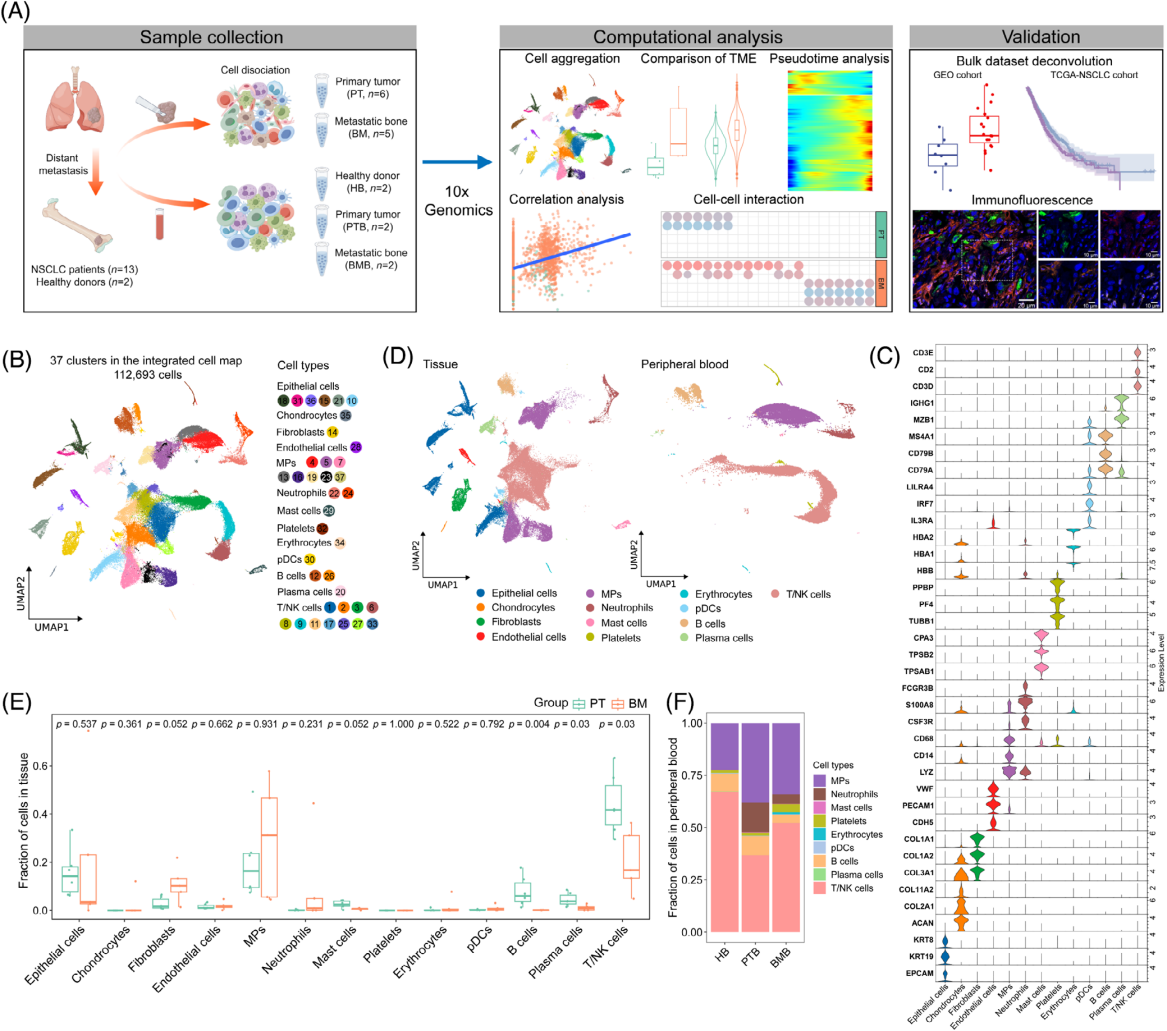
Single‐cell transcriptomic landscape of primary and bone metastatic NSCLC. (A) Workflow of sample collection and analysis in this study. (B) Uniform Manifold Approximation and Projection (UMAP) plot revealing the integrated cell map, with 37 cell clusters of 13 annotated cell types. Each dot presents an individual cell coloured by clusters. MPs, mononuclear phagocytes; pDCs, plasmacytoid dendritic cells; NK, natural killer. (C) Violin plot showing the scaled expression of representative marker genes across cell types. (D) UMAP plot showing the distinct cell composition split by tissue and peripheral blood sites in patients coloured by cell types. (E) Boxplot showing the cell type abundance in primary tumours (PT) and bone metastases (BM). (F) Fraction of immune cell types in peripheral blood from normal samples (HB), primary tumour (PTB) and bone metastases (BMB). NSCLC, non‐small cell lung cancer.

To characterize the distinct microenvironment in the BM of NSCLC, transcriptome profiles were generated by scRNA‐seq of 11 tissues from PT (*n* = 6) and BM (*n* = 5) and six peripheral blood samples from PT (PTB, *n* = 2), BM (BMB, *n* = 2), as well as normal samples (HB, *n* = 2), collected from 13 NSCLC patients and two healthy donors. An overview of our single‐cell analysis is shown in Figure 1A. Following standard processing and quality control, single‐cell data in a total of 112,693 cells were retained for downstream analysis (Figure 1B, Figure S1A). These cells were catalogued into 37 clusters of 13 major cell types, including epithelial cells, chondrocytes, fibroblasts, endothelial cells (ECs), mononuclear phagocytes (MPs), neutrophils, mast cells, platelets, erythrocytes, plasmacytoid dendritic cells (pDCs), B cells, plasma cells and T/NK cells (Figure 1B,C, Figure S1B,C, Table S2). The cell clusters presented significant variances in enrichment across samples obtained from tissues and peripheral blood (Figure 1D and Figure S2A–C). The fraction of fibroblasts in BM was higher than that in PT, whereas mast cells, B cells, plasma cells and T/NK cells were less infiltrated in BM than in PT (Figure 1E, Figure S2C,D). Additionally, in peripheral blood, the BMB group had a relatively lower proportion of B cells and T/NK cells than the HB group (Figure 1F, Figure S2C,D). The results indicated a lower level of adaptive immune infiltration in bone‐metastatic lesions of NSCLC.

### 2.2 Senescence state of cancer cells in bone metastasis of NSCLC and the contribution to tumorigenesis

**FIGURE 2:**
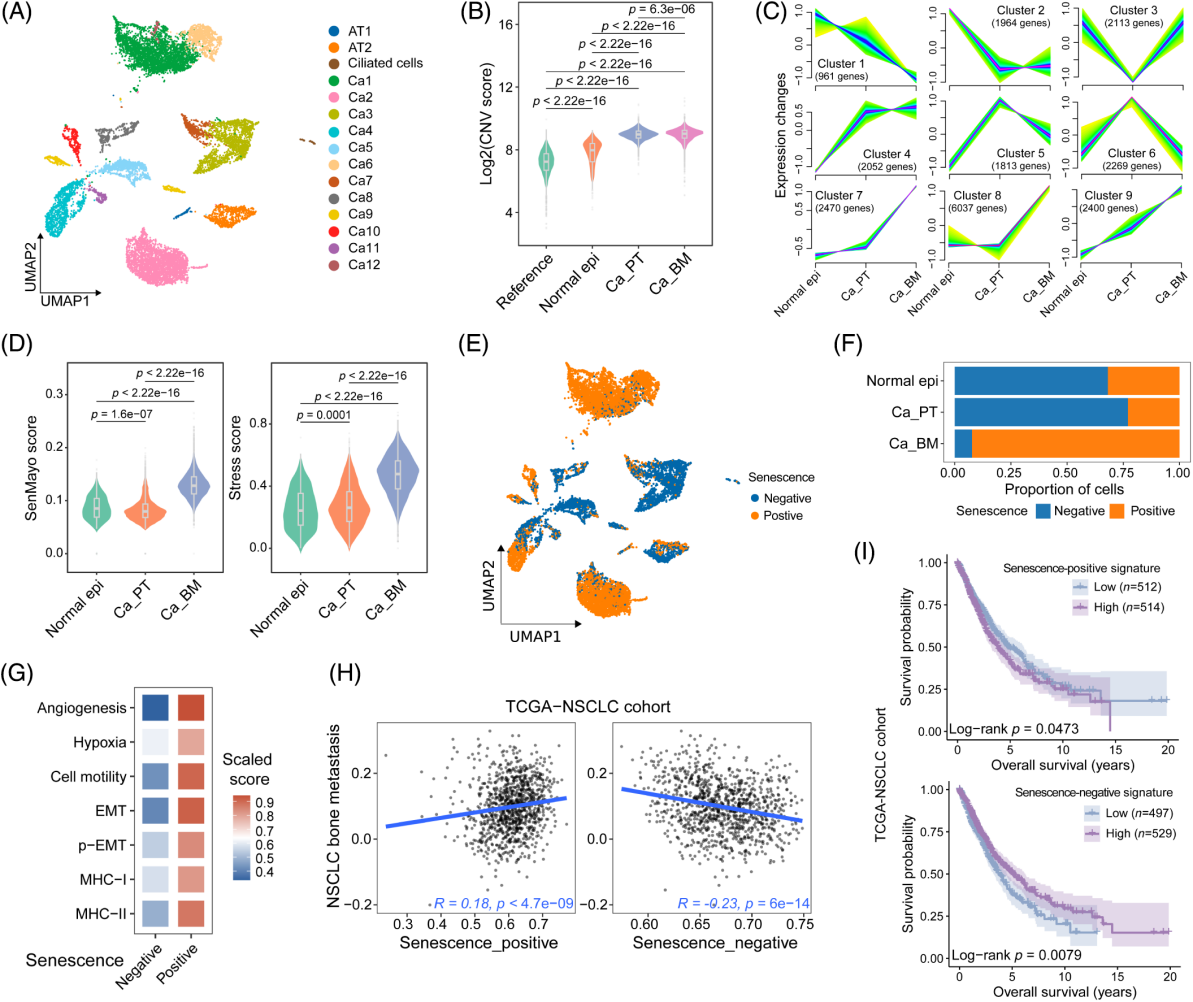
Senescent cancer cells with increased malignant properties in bone metastases of NSCLC. (A) UMAP plot of 13,189 epithelial cells clustered by annotated cell types. (B) Violin‐boxplot showing copy number variation (CNV) score across reference, normal epithelial cells, and malignant epithelial cells from PT (Ca_PT) and BM (Ca_BM). (C) Gene clustering by the mFuzz analysis to estimate the relative expression changes across normal epithelial cells, Ca_PT and Ca_BM. (D) Violin‐boxplots displaying the SenMayo and stress scores of normal epithelial cells, Ca_PT and Ca_BM. (E) UMAP plot of epithelial cells coloured by senescence‐negative and ‐positive clusters. (F) Fraction of senescence‐negative and ‐positive epithelial cells across the groups. (G) Heatmap showing the scaled score of interested signatures in malignant cells according to their senescence status. (H) Spearman correlation of the senescence‐negative and ‐positive signatures with NSCLC bone metastasis assessed by the “VICENT_METASTASIS_UP” signature from MSigDB. (I) Kaplan–Meier plots showing the difference in survival probability (%) in patients with high versus low scores of the senescence signatures according to the optimal cutoff based on the TCGA‐NSCLC cohort. NSCLC, non‐small cell lung cancer.

The epithelial cells were proportioned into 22 clusters, which were annotated as alveolar type I (AT1), alveolar type II (AT2), ciliated cells and cancer cells (Ca) based on the marker genes and sample distribution (Figure 2A, Figure S3A–D, Table S3). The inferred copy number variation (CNV) profile as well as the score related to the occurrence of NSCLC also distinguished cancer cells from non‐malignant cells (Figure S3E–G, Table S5). Additionally, the cancer cells from BM sites exhibited higher CNV scores compared to those from PT tissues (Figure 2B).

To explore the transcriptional changes associated with NSCLC progression, the mFuzz soft‐clustering analysis was performed (Figure 2C). Gene expression with an upward trend from primary malignant cells to metastatic malignant cells (Cluster 7–9) was associated with cytokine production, cellular stress, or aging‐related pathways (Figure 2C, Figure S4A, Table S4). To elucidate the engagement of cellular senescence, we employed the SenMayo^17^ and Fridman_up^18^ gene sets to assess the senescence state of epithelial cells in our study (Table S5). BM‐derived malignant cells presented higher stress and senescent scores than malignant cells from PT and normal epithelial cells (Figure 2D, Figure S4B). Therefore, malignant cells of NSCLC developed a senescent state induced by stress conditions during bone metastasis. We then divided all the epithelial cells into senescence‐positive and ‐negative cells, according to the optimal threshold of senescence activity based on the SenMayo signature (Figure 2E, Figure S4C), which were validated by canonical markers (Figure S4D). A shift towards the G1 phase occurred within the senescence‐positive cells, consistent with cell cycle arrest (Figure S4E). The frequency of senescence‐positive cells was considerably higher in BM‐derived malignant cells compared to primary malignant cells or normal epithelial cells (Figure 2F). According to enrichment analysis, senescence‐positive malignant cells expressed genes involved in inflammatory response, T cell activation, positive regulation of angiogenesis, positive regulation of cell motility and bone resorption, whereas the genes overexpressed in senescence‐negative malignant cells were related to cell cycle transition, biosynthetic metabolism process, and telomere maintenance (Figure S4F). We further explored the association of cellular senescence with metastasis‐related signature scores. Senescence‐positive cancer cells presented remarkably higher scores of angiogenesis, hypoxia, cell motility, complete epithelial‐mesenchymal transition (EMT) and partial EMT (p‐EMT) signatures than senescence‐negative cancer cells, which were validated in the TCGA‐NSCLC cohort, suggesting higher metastasis potential (Figure 2G, Figure S4G, Tables S5 and S6). Notably, senescence‐positive cancer cells also had higher scores of major histocompatibility complex class I and II (MHC‐I and MHC‐II) signatures, indicating enhanced immunogenic features (Figure 2G, Figure S4G).^19^ Based on the TCGA‐NSCLC cohort, we also observed that the senescence‐positive signature was significantly correlated to the bone metastasis feature and poor overall survival of patients (Figure 2H,I, Tables S5 and S6). Whereas patients with a high senescence‐negative signature score were less prone to bone metastasis, and undergoing favourable overall survival (Figure 2H,I, Tables S5 and S6). Overall, our results revealed evident senescence of malignant cells in the BM of NSCLC.

### 2.3 Senescent endothelial cells undergoing a mesenchymal transition in the bone metastatic environment

**FIGURE 3:**
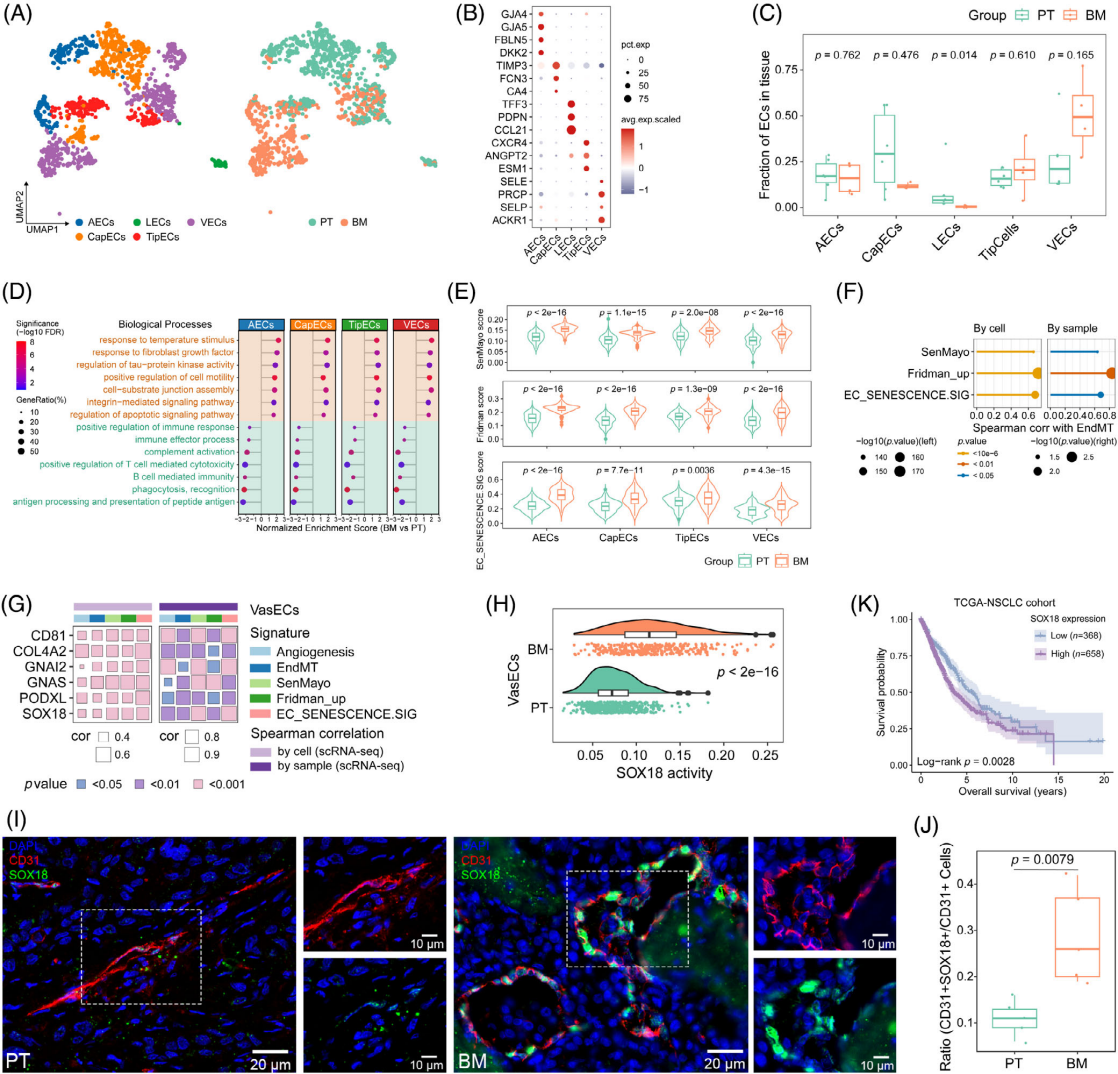
Transcriptional landscape of endothelial cells in primary tumours (PT) and bone metastases (BM). (A) UMAP plot of 1147 subclustered endothelial cells coloured by annotated cell types (left panel) and tissue origin (right panel). (B) Dot plot showing the scaled expression of representative marker genes in the annotated endothelial cells. (C) Boxplot indicating the proportion of annotated cell types in PT and BM. (D) Gene Set Enrichment Analysis of selected biological process terms regarding the differentially expressed genes between PT and BM across the vascular endothelial cells (VasECs). (E) Violin‐boxplot displaying senescent scores of endothelial cells in PT and BM. (F) Lollipop plot illustrating the Spearman correlation of the EndMT signature with the senescent signatures by cells (left panel) and by samples (right panel) in VasECs. EndMT, endothelial‐mesenchymal transition. (G) Heatmap illustrating the Spearman correlation of selected genes with interested signatures by cells (left panel) and by samples (right panel) in VasECs. (H) Raincloud plot showing the transcription factor activity level of SOX18 in VasECs from PT and BM. (I‐J) Representative immunofluorescence images (I) and boxplot (J) showing SOX18 expression level in PT and BM. Scale bar: 20 μm (200×) and 10 μm (400×). (K) Kaplan–Meier plot showing the difference in survival probability (%) in patients with high versus low expression of SOX18 according to the optimal cutoff based on the TCGA‐NSCLC cohort. NSCLC, non‐small cell lung cancer.

The qualified ECs were annotated into 5 subtypes, namely artery ECs (AECs), capillary ECs (CapECs), lymphatic ECs (LECs), TipECs and vein ECs (VECs), based on their marker genes (Figure 3A,B, Figure S5A,B, Table S7). BM presented less abundance of LECs compared to PT (Figure 3C, Figure S5C,D). We then performed Gene Set Enrichment Analysis (GSEA) to investigate the functional heterogeneity of ECs between BM and PT. BM‐derived vascular endothelial clusters (VasECs; including AECs, CapECs, TipECs and VECs) demonstrated upregulation of genes involved in cell motility, integrin‐related and apoptotic pathways (Figure 3D), indicative of cellular senescence according to a recent study.^20^ The cellular senescence in BM‐derived VasECs was confirmed by increased canonical markers linked to DNA damage response (CDKN1A, TP53 and H2AFX) and enrichment of oxidative stress‐induced senescence based on the REACTOME database (Figure S5E,F). We then further assessed the senescence‐associated secretory phenotype (SASP) feature of ECs using the aforementioned SenMayo and Fridman_up signatures, together with a third signature called EC_SENESCENCE.SIG that was recently identified for the determination of pan‐cancer endothelial senescence.^20^ VasECs displayed relatively higher senescence scores compared to the LECs across the three signatures (Figure S5G). Of note, BM‐derived VasECs broadly exhibited upregulation of senescence scores compared to those from PT (Figure 3E).

Regarding the functional implications of senescence in VasECs, we observed positive associations of senescence scores with EndMT^21^ and angiogenesis (Figure 3F, Figure S6A, Table S5). Intriguingly, BM‐derived VasEC clusters showed an increase in the expression of mesenchymal markers (VIM, SPARC and SERPINE1), indicating an aggravated EndMT feature (Figure S6B). Additionally, we observed a stepwise upregulation of mesenchymal markers during the trajectory pseudotime (Figure S6C,D), corroborating the notion that the ECs in BM are a result of cells undergoing EndMT rather the mesodermal precursors, which was consistent with the results in another study.^22^ We verified the overexpression of SERPINE1 in ECs from BM by mIF staining (Figure S6E,F), which was previously reported to characterize ECs with a high senescent status.^23^ Given the broad senescence state in BM‐derived VasECs, we further identified 186 common genes upregulated across the four clusters in BM compared to PT (Figure S7A), with six genes positively associated with the signatures of angiogenesis, EndMT and cellular senescence, which were validated in the TCGA‐NSCLC cohort (Figure 3G, Figure S7B). Of note, SOX18 was specifically expressed in ECs (Figure S7C), with the TF activity showing significant upregulation in VasECs from BM compared to PT (Figure 3H). mIF analysis further validated the activation of SOX18 in ECs from BM patients (Figure 3I,J). Additionally, SOX18 overexpression was related to a poor response to immune checkpoint blockade (ICB) treatment and disease progression post‐treatment (Figure S7D), as well as unfavourable overall survival for NSCLC patients (Figure 3K). In summary, these results suggested that BM‐derived vascular ECs manifested senescence, accompanied by the EndMT process and sprouting angiogenesis, which was characterized by the excessive expression of SOX18.

### 2.4 Increased CD4Tstr cells and implications in the bone‐metastatic microenvironment

**FIGURE 4:**
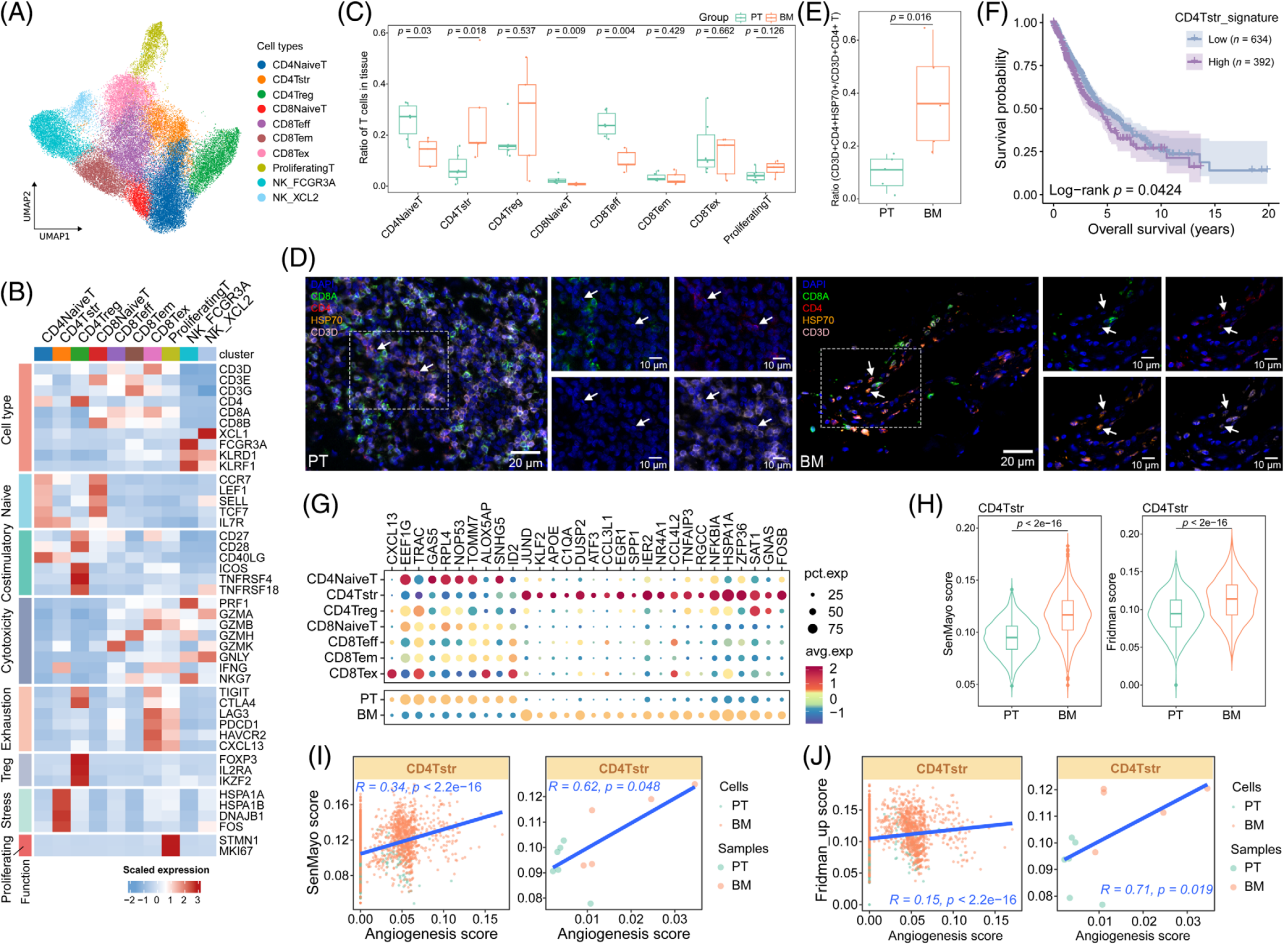
CD4Tstr cells exhibit pronounced senescent features in the bone‐metastatic microenvironment. (A) UMAP plot of 45,603 subclustered T/NK cells coloured by annotated cell types. (B) Heatmap illustrating the expression of selected signatures in annotated subtypes. (C) Boxplot indicating the proportion of annotated cell types in PT and BM. (D‐E) Representative immunofluorescence images (D) and boxplot (E) showing the expression level of CD4Tstr cells (positive for CD3D, CD4, and HSP70) in PT and BM. Scale bar: 20 μm (200×) and 10 μm (400×). (F) Kaplan–Meier plot showing the difference in survival probability (%) in patients with high versus low scores of the CD4Tstr signature according to the optimal cutoff based on the TCGA‐NSCLC cohort. (G) Dot plot displaying the expression of selected genes across T‐cell subclusters and sample groups. (H) Violin‐boxplots displaying senescent scores in CD4Tstr cells from PT and BM. (I) Spearman correlation of the SenMayo signature with the angiogenesis signature by cells (left panel) and by samples (right panel) in CD4Tstr cells. (J) Spearman correlation of the Fridman_up signature with the angiogenesis signature by cells (left panel) and by samples (right panel) in CD4Tstr cells. PT, primary tumour; BM, bone metastases; NSCLC, non‐small cell lung cancer.

The T/NK cells were subclustered into 10 populations based on canonical markers (Figure 4A,B, Figure S8A,B, Table S8). CD4Tstr cluster, which was previously identified in recent studies,^24^, ^25^ highly expressed heat shock and stress‐related genes, including HSPA1A, HSPA1B, DNAJB1 and FOS. Intriguingly, the CD4Tstr cluster from tissues also exhibited remarkable features of resident, angiogenesis and inflammation, which may be associated with the metastasis of NSCLC (Figure S8C, Table S5). Within tissues, BM exhibited higher fractions of CD4Tstr cells and less infiltration of the CD8Teff cluster (Figure 4C). A higher ratio of CD4+/CD8+ T cells was observed in BM compared to PT, which was a reversed ratio difference between metastatic and normal peripheral blood mononuclear cells (Figure S9A). The preferential infiltration of the CD4Tstr cluster in BM was validated by the mIF staining (Figure 4D,E). Notably, the elevated expression of the CD4Tstr signature was correlated with an unfavourable clinical outcome in the TCGA‐NSCLC cohort (Figure 4F, Table S6). Interestingly, the two naive T‐cell clusters exhibited lower levels of infiltration in BM compared to PT, indicating a relatively limited regenerative capacity of immune cells in the bone metastasis environment (Figure 4C and Figure S8D–F).

Regarding the functional heterogeneity, the T‐cell clusters in BM displayed more activities of stress, ECM remodelling, angiogenesis, inflammatory state and lipid metabolism than those in PT. Additionally, CD4Treg cells specifically exhibited heightened signals of Treg, costimulatory molecules and cytokine/cytokine receptors in BM, indicating intensified immunosuppressive capability in BM (Figure S9B). However, these functional disparities were not observed between the metastatic and primary environments of peripheral blood origin, indicating the contribution of tumour‐infiltrated immune cells to shape the environment in the metastatic niche (Figure S9C). According to the GSEA results, genes upregulated in BM were commonly related to response to unfolded protein, angiogenesis, aging and the p38MAPK cascade across the T‐cell clusters (Figure S9D). Based on the alterations of transcriptional profiles and T‐cell compositions, including decreased infiltration of NaiveT cells and increased CD4+/CD8+ T‐cell ratio, T lymphocytes in BM accorded a senescent phenotype.^26^, ^27^, ^28^ Comparing the DEGs among T‐cell clusters and the DEGs within T cells between PT and BM tissues, we observed the most overlap (*n* = 116) between the genes specifically expressed in CD4Tstr cells and the DEGs between tissues, indicating that these genes were responsible for the transcriptional heterogeneity between T cells from primary and metastatic tumours (Figure 4G, Figure S10A). Intriguingly, CD4Tstr cells displayed the highest senescence scores (SenMayo^17^ and Fridman_up^18^ signatures, Table S5) among the seven T‐cell clusters, while the two naive clusters were in the least senescent state (Figure S10B). We therefore focused on the implication of cellular senescence for the CD4Tstr cluster. Cellular senescence mainly occurred in the tissue‐derived CD4Tstr cluster (Figure S10C), with CD4Tstr cells in BM displaying more aggravated senescence than those in PT (Figure 4H). Whereas the senescence heterogeneity was not found between groups of peripheral blood origin (Figure S10D). The aggravated senescence state in BM‐derived CD4Tstr cells was validated by increased expression of canonical markers related to DNA damage response (TP53, CDKN1A and H2AFX) and decreased expression of costimulatory molecules (CD27 and CD28)^29^ (Figure S10E). Further, we observed positive associations of senescence scores with stress, inflammation, and angiogenesis scores in the CD4Tstr cluster (Figure 4I,J, Figure S10F,G). The findings revealed that the senescence of CD4Tstr cells in the tumour‐infiltrated bone environment, with the engagement of a pro‐inflammatory secretory phenotype, may promote angiogenesis to support tumour colonization.

### 2.5 Immunosenescent CD4Tstr state drives the bone metastasis process in NSCLC

**FIGURE 5:**
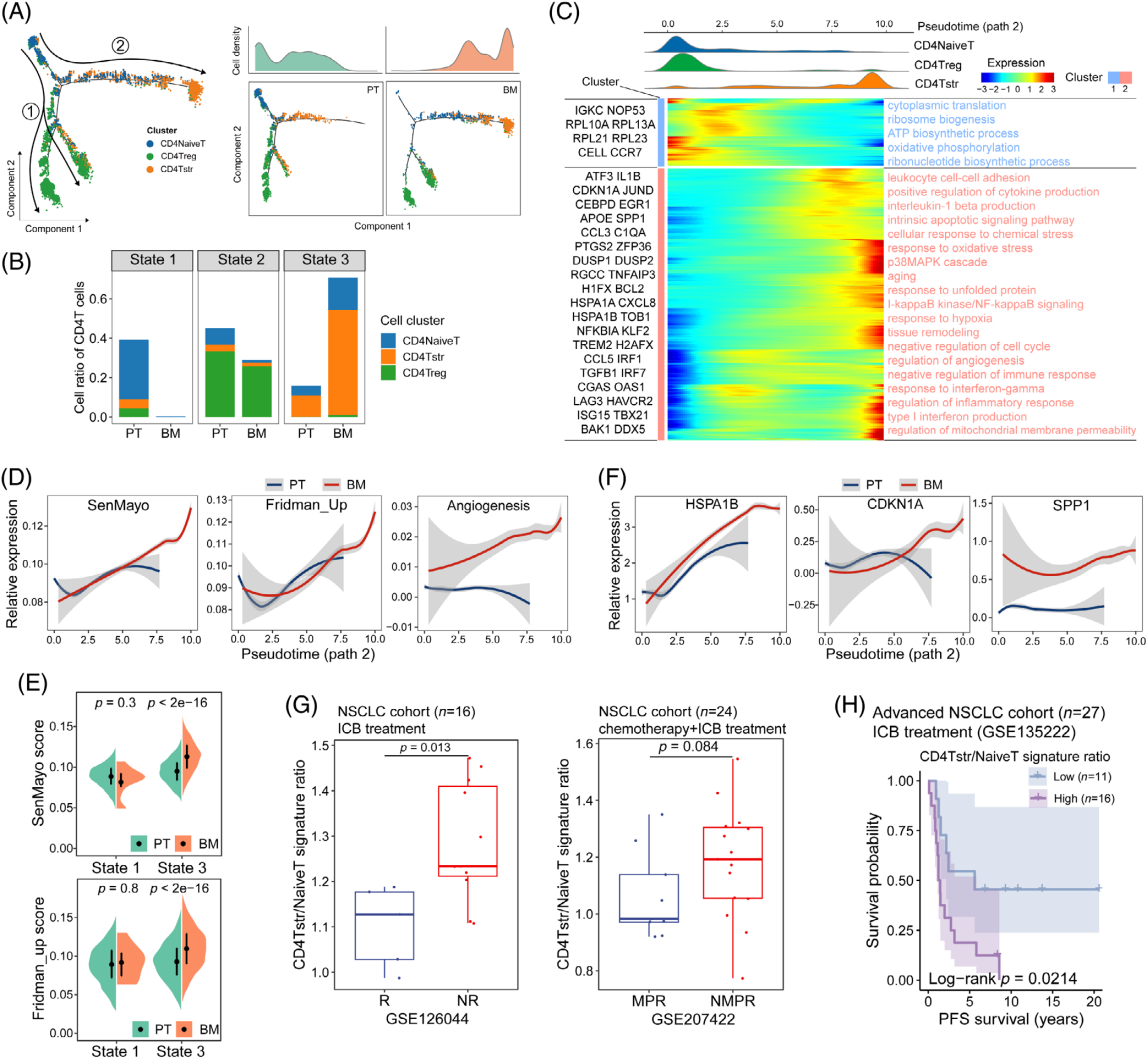
Development trajectory of CD4+ T cells in primary tumours (PT) and bone metastases (BM). (A) The pseudotime distribution of all (left panel) and split (lower‐right panel) CD4+ T cells coloured by cell subtypes. Cell density along the pseudotime trajectory in PT and BM (upper‐right panel). (B) Histogram showing the cell fraction of CD4+ T cells in PT and BM. (C) The cell distribution and gene expression changes of CD4+ T cells along the trajectory (path 2). (D) The dynamic changes of interested signatures along the pseudotime (path 2). (E) Split violin plots illustrating the scores of the senescent signatures in different states as indicated in Figure 5B. (F) The dynamic changes of selected genes along the pseudotime (path 2). (G) Boxplots displaying the CD4Tstr/NaiveT signature ratio in responders and non‐responders with chemotherapy and ICB treatments in independent NSCLC cohorts. R, response; NR, non‐response; MPR, major pathologic response; NMPR, non‐MPR. (H) Kaplan–Meier plot showing the difference in progression‐free survival (PFS) probability (%) in NSCLC patients receiving ICB treatment with high versus low CD4Tstr/NaiveT signature ratio according to the optimal cutoff in an independent cohort. ICB, immune checkpoint blockade; NSCLC, non‐small cell lung cancer.

To explore the dynamic immune states and cell transitions, we inferred the state trajectory in tumour‐infiltrated CD4+ T subtypes using Monocle2.^30^ The CD4+ T‐cell trajectory started with NaiveT cells and branched into two distinct paths, with path 1 ending in the Treg cells and path 2 ending in the Tstr cells, marking their divergent differentiation process (Figure 5A, Figure S11A). Therefore, cellular stress appears to be a separate process from the immunosuppression mediated by regulatory T cells. The cell trajectory was divided into 3 states, which have comparable fractions of cells (Figure S11B,C). The transition states along path 2 were related to cell origin, with early‐stage CD4+ T cells (state 1) mainly distributed in PT and the terminal ends of CD4+ T cells (state 3) predominant in BM, indicating a transition process from naive to stress states during metastasis (Figure 5A,B). Of note, the CD4Tstr cluster abundance was negatively associated with NaiveT fraction (Figure S11D).

The transcriptional changes along path 2 were characterized by the upregulation of genes (cluster 2) involved in response to oxidative stress (HSPA1B, JUN, TNFAIP3 and KLF2), p38MAPK cascade (DUSP1, ZFP36, IL1B and TREM2), aging (CDKN1A, PTGS2 H2AFX), cytokine production and chemotaxis (SPP1, EGR1, IL1B and CXCL8), and regulation of mitochondrial membrane permeability (BAX, BAK1), which were associated with cellular senescence (Figure 5C).^31^, ^32^ We then compared the alteration diversity of the senescence signatures between groups over pseudotime. BM revealed progressive upregulation of the SenMayo and Fridman_up scores along the transition trajectory, which were higher than those in PT in state 3 (Figure 5D,E), indicating a worsened senescent state during the progression of BM. In addition, the senescence‐related hallmark, angiogenesis, was observed to exhibit a steady increase in BM but only show minimal changes in PT along the development (Figure 5D). The expression of HSPA1B, CDKN1A, along with SPP1, an SASP factor, also displayed differential changes between BM and PT at the terminal stage of the path 2 trajectory (Figure 5F). SPP1 was found to be associated with angiogenesis score in CD4+ T cells (Figure S11E), which may be involved in tumour progression.^33^ Intriguingly, a high CD4Tstr to NaiveT signature ratio was related to a poor response to ICB and unfavourable progression‐free survival of NSCLC patients post‐treatment (Figure 5G,H, Figure S11F, Table S6). These findings revealed that increased cellular stress and immunosenescence, along with the pro‐angiogenesis of CD4+ T cells during tumour progression, may hinder immune‐based intervention strategies, hence supporting the bone metastasis of NSCLC.

### 2.6 SPP1 signalling induces distinct cell interactions in the bone metastatic environment of NSCLC

Ultimately, to decipher the crosstalk between cells in the bone metastatic microenvironment, we utilized CellChat^34^ to explore ligand‐receptor (LR)‐mediated intercellular interactions in PT and BM (Table S9). More frequent cell–cell interactions between cancer cells, T cells and VasECs were observed in BM (Figure S12A–C). Of note, senescent cancer cells exhibited greater contact with CD4Treg cells via the MHC‐II pathway compared to non‐senescent counterparts (Figure S12D). The cellular communication along with the increased Treg score in BM (Figure S9B) indicated senescent cancer cells in BM may induce more intensive immunosuppression signals via the antigen presentation process.

**FIGURE 6:**
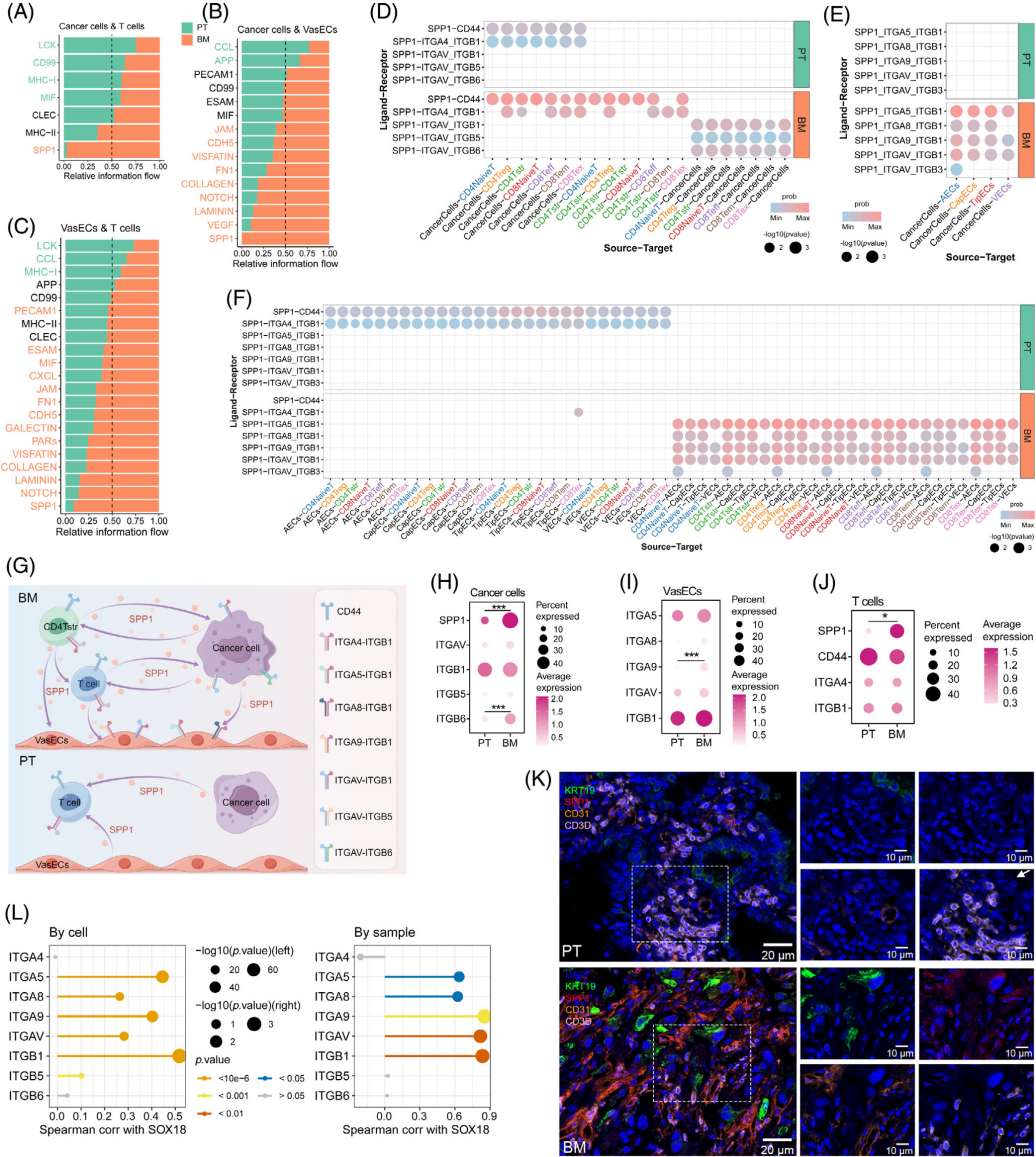
Cell–cell communication networks in primary tumours (PT) and bone metastases (BM). (A–C) Bar plots showing the fraction of relative information flow of signalling pathways in PT and BM between cancer cells and T cells (A), between cancer cells and VasECs (B), as well as between T cells and VasECs (C). (D–F) Dot plots showing significant ligand‐receptor pairs contributing to SPP1 signalling in PT and BM between cancer cells and T cells (D), between cancer cells and VasECs (E), as well as between T cells and VasECs (F). Dot colour and size represent the calculated communication probability and *p*‐value, respectively, determined from the one‐sided permutation test. (G) Inferred regulation network among cancer cells, T cells, and VasECs. (H‐J) Dot plot displaying the expression of selected genes in cancer cells (H), VasECs (I) and T cells (J) from PT and BM. (K) Representative immunofluorescence images showing SPP1 expression in PT and BM. Scale bar: 20 μm (200×) and 10 μm (400×). (L) Lollipop plot illustrating the Spearman correlation of SOX18 with receptors by cells (left panel) and by samples (right panel) in VasECs.

Strikingly, we found that the SPP1 pathway was mainly activated in the interactions between the three cell types of BM origin, which was rarely found in those of PT origin (Figure 6A–C). Likewise, senescence‐positive cancer cells tended to interact with T cells or VasECs via the SPP1 pathway more intensively than senescence‐negative counterparts (Figure S13A). Additionally, the interactions through the SPP1‐CD44 and SPP1‐ITGA4_ITGB1 pairs between cancer cells and T cells occurred more frequently in BM than in PT and were also more prevalent in senescence‐positive cancer cells compared to senescence‐negative counterparts (Figure 6D, Figure S13B). Notably, CD4Tstr, the subtype characterized by senescence, could signal to CD4Treg cells through the SPP1‐CD44 and SPP1‐ITGA4_ITGB1 pairs in BM, implying the involvement of the two pairs in senescence‐related immunosuppressive communications (Figure 6D). Cell communication via the SPP1‐CD44 pair has been previously identified to be aggravated in aging conditions.^35^ Additionally, SPP1 as a ligand could induce communications of pan T‐cell subtypes to cancer cells specifically in BM or to senescent cancer cells via adhesive receptors ITGAV_ITGB1, ITGAV_ITGB5 and ITGAV_ITGB6 (Figure 6D, Figure S13B). Similarly, the ligand‐receptor pairs SPP1‐ITGA5_ITGB1, SPP1‐ITGA8_ITGB1, SPP1‐ITGA9_ITGB1, as well as SPP1‐ITGAV_ITGB1, were also identified in the communications of both cancer cells and T cells with VasECs specifically in BM, as well as in the communications of senescent cancer cells with VasECs (Figure 6E,F, Figure S13C). An interaction network consisting of seven ligand‐receptor pairs representative of the SPP1 signalling pathway was identified to induce the communications among cancer cells, T cells and VasECs in BM of NSCLC (Figure 6G). The interaction network with specificity to BM and senescent cancer cells was mainly due to the significant upregulation of SPP1 ligand in BM‐derived or senescent cancer cells and T cells from BM (Figure 6H–J, Figure S13D). mIF results also observed a more prevalent staining pattern of SPP1 in T cells and cancer cells from BM (Figure 6K). Moreover, upregulation of the ligand‐receptor pairs was significantly related to bone metastasis and unfavourable overall survival of NSCLC patients (Figure S13E,F, Table S5). Since we observed positive associations of the receptors with SOX18 in VasECs, the cellular communication may activate SOX18 to promote metastasis (Figure 6L). Taken together, the communication network between cancer cells, T cells and vascular ECs, regulated by ligand‐receptor pairs representative of SPP1 signalling, may be an important mechanism responsible for bone metastasis and the poor prognosis of NSCLC patients.

## 3 DISCUSSION

Since the bone microenvironment has recently been recognized to endow the secondary dissemination of metastatic cancer cells,^36^ it is imperative to explore bone metastatic seeding from primary lesions to prevent the eventual multi‐organ metastases and cancer‐related deaths. In this study, we uncovered a senescent environment in the BM of tissue origin in NSCLC, which was induced by tumour‐related cellular stress and inflammation rather than age since the latter was comparable between the participants of PT and BM.

Metastases frequently colonize bones rich in red marrow and trabecular bone, since the higher rates of bone turnover and vascularization present in these sites are favourable for tumour cell seeding and growth.^37^ EndMT is a distinct biological process regulated by angiogenic sprouting, promoting migration and invasion into extracellular matrices of diverse composition.^38^ Previous studies reported that the EndMT process of vascular ECs could be induced by stress‐related senescence under radiation treatment.^23^ Our study identified an aggravated senescent phenotype of vascular ECs in the BM of NSCLC, which was likewise associated with the EndMT feature. Additionally, we identified SOX18 upregulated in senescent ECs as a putative bone metastasis‐related molecule for NSCLC with the involvement of the EndMT process. Similarly, Luo et al. recently reported increased activity of SOX18 in the cancer‐associated fibroblasts derived from EndMT.^39^ SOX18‐induced modulation of endothelial barrier integrity is crucial for cancer progression and metastasis.^40^ Blockade of SOX18‐dependent interactions, on the other hand, reduced the tumour vascular density and potently suppressed tumour metastasis of melanomas and breast cancer.^41^, ^42^ Based on the results, we suggested SOX18 as a therapeutic marker for advanced NSCLC to inhibit the metastatic spread to bone and improve the outcome of patients.

Circulating T‐cell immunosenescence was previously observed in advanced NSCLC patients.^43^ However, little is known regarding the presence of senescent T cells in the TME and their contribution to distant metastasis. We here found an aggravated senescent immune phenotype of tumour‐infiltrating T cell populations in NSCLC BM, characterized by decreased fractions of NaiveT cells and enriched CD4Tstr cells. Of note, CD4Tstr cells, which contribute predominantly to the transcriptional heterogeneity of T cells between PT and BM, display a particularly pronounced senescent condition, which was positively associated with angiogenesis. Angiogenic T cells, characterized by the secretion of angiogenic molecules, were previously identified to present senescent features under a chronic inflammatory environment.^44^ Intriguingly, a high CD4Tstr/NaiveT state ratio is found to be associated with an unfavourable response to ICB therapy and disease progression post‐treatment in NSCLC. A recent study also observed significant enrichment of Tstr cells and overexpression of stress‐related genes in nonresponsive tumours following ICB therapy across cancer types.^25^ Further verification of the mechanism of T‐cell senescence in NSCLC BM may aid in improving the antitumor immune impairment for advanced NSCLC patients.

In the senescent environment of BM, a complex cell communication network involving cancer cells, T cells and vascular ECs, induced by SPP1 signalling, was uncovered, indicative of enhanced neovascularization and immunosuppressive capability in the metastatic niches. During peritoneal metastasis from colorectal cancer, senescent PT cells produce angiogenesis‐related genes to support neovascularization, facilitating pre‐metastatic niches in distant sites.^45^ Another study also reported that the immunosenescence‐related disproportion may result in inflammatory senescence of vascular ECs.^46^ On the other hand, SASP factors from ECs contribute to the bone homing and aggressiveness of cancer cells.^47^ Ablation of senescent cells induces an immunostimulatory phenotype of T cells and a decrease in the tumour vascular network.^48^ In lung cancer cells, SPP1 interaction with ανβ3 integrin was found to increase their preferential migration to the bone matrix.^49^ A recent study of colorectal cancer highlighted the infiltration of senescent tumour cells around SPP1+ macrophages with remarkable SASP features.^50^ Our study, however, observed upregulation of SPP1 in senescent T cells and cancer cells from BM, characterizing the association of the ligand‐receptor axes of the SPP1 pathway with senescence, immunosuppression and angiogenesis in BM of NSCLC patients. The EndMT process observed in the VasECs in our study was reported to occur under SPP1 pathway‐mediated cell‐to‐cell communication.^39^ Considering the contribution of the SPP1 pathway to unfavourable overall survival for NSCLC patients, therapy targeting SPP1‐related cell–cell interactions may provide insights into the inhibition of the bone metastasis of NSCLC.

Our study has some limitations. Firstly, due to the difficulty in the sample acquisition of BM via surgical resection, the study collected a limited sample size of resected bone metastatic tissues, with PT the reanalyzed data of previously published cohorts. Secondly, because of the sample‐processing difficulty of the mixed metastatic soft tumours and sclerotic bone tissues, our data may not reflect the entire ecosystem of metastatic NSCLC to the bone. Besides, further experiments need to be conducted to clarify the question of whether senescent T cells are the consequence or the cause of the metastasis.

In conclusion, our study at high resolution uncovered a senescent microenvironment in the metastatic bone niche of NSCLC characterized by aggravated metastatic properties of cancer cells, vascular ECs undergoing EndMT, and pro‐angiogenic CD4Tstr cells. The intratumoral crosstalk among T cells, vascular ECs, and malignant cells induced by the SPP1 pathway with subsequent activation of SOX18 in vascular ECs is also highlighted, helping a deeper understanding of the mechanisms underlying the senescent microenvironment in metastatic NSCLC. Although further functional validation of these analyses is warranted, our dataset can serve as a valuable resource for the design of targeted therapies and immunotherapeutic approaches for advanced NSCLC.

## 4 MATERIALS AND METHODS

### 4.1 Human specimens

Five patients pathologically diagnosed with BM of NSCLC were enrolled in this study for single‐cell sequencing. Tissue samples of the metastatic bone tumours were collected by surgical resection. The histopathology of tumours was confirmed via haematoxylin–eosin staining by senior pathologists. Additionally, four peripheral blood samples were obtained before surgery, two of which were from the five metastatic patients and the remaining from two patients with primary lung cancer. This study accords with the Declarations of Helsinki. All research protocols involving human samples were approved by the Ethics Committee of the First Affiliated Hospital of Fujian Medical University. Written informed consent was provided by all participants.

For comparison, we downloaded the single‐cell datasets of six tissue samples from primary NSCLC in Lambrechts' study (E‐MTAB‐6149 and E‐MTAB‐6653)^51^ and datasets of two peripheral blood samples from healthy donors in Zhu's study (GSE190510).^52^ The included patients with primary NSCLC (range, 55–86 years) and BM (range, 28–68 years) have a similar median age of 65 and 64 years, respectively. The characteristics of all the participants are summarized in Table S1.

### 4.2 Single‐cell suspension preparation, library construction and sequencing

Resected fresh tissue samples were digested into cell suspensions using gentleMACS (Miltenyi Biotec, Germany) at 37°C. After filtered through a 40‐μm sterile strainer (Miltenyi), cells were resuspended in red blood cell lysis solution (Singleron, China) to remove red blood cells. Peripheral blood mononuclear cells were isolated by density gradient centrifugation using Ficoll‐Paque Plus medium (GE Healthcare) and washed with Ca/Mg‐free PBS. Red blood cell lysis buffer (Singleron) was then added to remove the red blood cells. The cell viability was over 85%, as determined by staining with 0.4% trypan blue (Beyotime, China). The single‐cell suspensions were then adjusted to a concentration of roughly 700–1200 cells/μl and loaded onto a 10X Genomics Chromium instrument using the 10X Genomics Chromium Single‐Cell 3′ kit (V3) following the manufacturer's instructions. The subsequent steps of cDNA amplification and library construction were performed according to the standard manufacturer protocols. Completed libraries were sequenced on an Illumina NovaSeq 6000 system.

### 4.3 Single‐cell data processing and data integration

The raw sequencing data underwent alignment (reference genome GRCh38), barcode assignment, and unique molecular identifier (UMI) counting using the CellRanger software (version 6.0.0, 10X Genomics). Based on the Scanpy package (version 1.8.2),^53^ low‐quality cells for each sample dataset were filtered out following the criteria: gene count less than 200 or top 2% gene count, top 2% UMI count, mitochondrial content >10%, genes expressed in less than five cells. The remaining count matrix was subjected to log normalization, dimensionality reduction, and clustering with principle component analysis (PCA) and was visualized by utilizing Uniform Manifold Approximation and Projection (UMAP). The batch effect between samples was corrected using the Harmony (version 0.1.1) method,^54^ a superior iterative clustering approach to align cells from different batches. In the identification process of the subpopulations for ECs and T/NK cells, the Harmony was performed with default parameters using the top 50 PCA components among samples.

### 4.4 Differentially expressed genes (DEGs) and cell type annotation

Genes expressed in more than 10% of the cells in a cluster and with an average logarithm fold‐change of expression greater than 0.25 were selected as DEGs using the FindAllMarkers() function in the Seurat package (version 3.1.2).^55^ To further identify sub‐clusters, the cells in each major cell type were subjected to normalization, dimensionality reduction, and clustering as abovementioned. DEGs between one and other clusters were defined using the FindMarkers() function. Genes functionally related to cell subtype or state via literature review were used to annotate the subclusters in each major cell type. Multiplets were removed by the DoubletFinder package (version 2.0.3).^56^

### 4.5 Identification of malignant cells with CNV analysis

To identify malignant epithelial cells, chromosomal CNV for individual epithelial cells was determined using the InferCNV package (version 1.8.1)^57^ with default parameters. Epithelial cells were used for the observations, with lymphocytes (T and B cells) used as the reference.

### 4.6 Pseudotime analysis

The cell developmental trajectory was inferred using the Monocle2 package (version 2.99.3)^30^ with default parameters to determine the potential process of functional changes and lineage differentiation of cells. The integrated gene expression matrices of indicated subpopulations extracted from Seurat were converted to the CellDataSet object. Then, the data were subjected to dimension reduction based on the DDRTree method and cell ordering according to the significant differentially expressed genes between subclusters, followed by differentiation trajectory inferring.

### 4.7 Transcription factor regulatory network analysis

The pyscenic package (v0.11.0)^58^ was utilized to identify the transcription factor network based on the scRNA expression matrix and transcription factors in the AnimalTFDB database. The gene co‐expression network was identified via GRNBoost2. Regulons were identified using RcisTarget. Regulon activity for each single cell was determined using the AUCell function.

### 4.8 Signature scoring

Marker genes associated with specific signatures were curated from the literature (Table S5), to evaluate the potential biological functions of interested cells. For single‐cell data, we calculated the scores of functional signatures using the UCell package (version 2.6.0)^59^ at the individual‐cell level and their average at the sample level if needed. For bulk sequencing data, the ssGSEA method was used to calculate the functional scores of the signatures in individual samples.

### 4.9 Pathway enrichment analysis

We performed enrichment analysis using the clusterProfiler package (version 4.6.2).^60^ Pathways with adjusted P values <0.05 using the BH method, were considered significant.

### 4.10 Pattern clustering analysis

mFuzz package (version 2.58.0)^61^ was used to cluster gene expression patterns among multiple groups based on the genes with differential expression between groups.

### 4.11 Cell–cell interaction analysis

CellChat (version 1.5.0)^34^ was utilized to determine the intercellular communication network and identify the communicating molecules between different cell subtypes. Normalized count data were input to create the CellChat object and underwent calculation of communication probability, strength number of significant cellular communications and the contribution of each ligand‐receptor pair to the signalling pathway, with recommended parameters. The communicated difference in ligand‐receptor pairs among tumour ecosystems between groups was calculated based on a permutation test, with a *p* value <0.05 regarded as significant.

### 4.12 Analyses based on public datasets

Bulk transcriptome datasets of PT samples with treatment were obtained from the Gene Expression Omnibus (GEO) database under accession numbers GSE126044, GSE135222 and GSE207422. The transcriptomic expression and clinical data of TCGA‐LUAD and TCGA‐LUSC cohorts were obtained from The Cancer Genome Atlas (TCGA, https://portal.gdc.cancer.gov/) and merged into an NSCLC cohort (TCGA‐NSCLC) to evaluate the prognostic value of indicated genes or signatures for NSCLC patients, with the batch effect removed by the “sva” package (version 3.46.0).^62^ The tumour samples were classified into two subgroups using the “survminer” package (version 0.4.9) with the recommended cutoff value. The survival curves were fitted using the Kaplan–Meier method in the “survival” package (version 3.5–5), with the survival difference determined by the log‐rank test.

### 4.13 Multiplex immunofluorescence (mIF) staining

mIF staining was performed using the Opal Multiplex IHC kit (Akoya Biosciences, NEL861001KT). After deparaffinization, rehydration, and antigen retrieval by microwave, the tissue slides were incubated with primary antibodies targeting CD3D (1:200; 16669‐1‐AP; Proteintech, Wuhan, China), CD4 (1:200; 67786‐1‐Ig; Proteintech), CD8A (1:400; 66868‐1‐Ig; Proteintech), HSP70 (1:100; 66183‐1‐Ig; Proteintech), SOX18 (1:100; sc‐166025; Santa Cruz Biotechnology, Dallas, TX, USA), CD31 (1:200; 66065‐2‐Ig; Proteintech), SERPINE1 (1:100; sc‐5297; Santa Cruz Biotechnology), KRT19 (1:200; 10712‐1‐AP; Proteintech) and SPP1 (1:100; 22952‐1‐AP; Proteintech). Then, the slides were subjected to incubation with secondary antibodies and corresponding reactive Opal fluorophores. Following each tyramide signal amplification operation, the slides underwent microwave heat treatment. Nuclei acids were stained with 4′,6‐diamidino‐2‐phenylindole (DAPI) after all the antigens had been labelled. As a negative control, tissue slides were incubated with primary and secondary antibodies without fluorophores to assess autofluorescence. The Vectra Polaris multispectral Imaging System (Akoya Biosciences) was used to image the tissue slides.

### 4.14 Statistical analysis

All the statistical analyses were performed using the R software (version 4.2.3). The unpaired Student's t‐test and Wilcoxon rank‐sum test were utilized to compare the differences between the two groups. Correlations between data were explored using Spearman's correlation test. A *P* value <0.05 was considered statistically significant.

## AUTHOR CONTRIBUTIONS

S.W. and L.A. performed bioinformatic analyses and wrote the original draft. H.L. and H.W. conducted sample collection and validated the results. S.L., H.Z. and T.M. performed data curation and visualization. Z.W., F.L. and R.S. patriated in the experiments. G.Z., J.L. and X.S. conceived this study and revised the manuscript. All authors approved the final version of the manuscript.

## CONFLICT OF INTEREST STATEMENT

The authors declare no conflict of interest.

## Supporting information


**Data S1.** Supplementary figures.

**Table S1.** Characteristics of the NSCLC patients included for scRNA‐seq.

**Table S2.** Differentially expressed genes across cell clusters in the integrated cell map.

**Table S3.** Differentially expressed genes across cell clusters of epithelial cells.

**Table S4.** Gene ontology terms enriched in clusters of epithelial cells by mfuzz method.

**Table S5.** Gene signatures curated form literature in this study.

**Table S6.** Gene signatures identified in this study.

**Table S7.** Differentially expressed genes across cell clusters of endothelial cells.

**Table S8.** Differentially expressed genes across cell subtypes of lymphoid cells.

**Table S9.** Cell communications between cell clusters by Cellchat.

## Data Availability

The data that support the findings of this study are openly available in Genome Sequence Archive in National Genomics Data Center at https://ngdc.cncb.ac.cn/gsa/, reference number HRA007575.
