# Supplementary material for: Single‐cell transcriptomic analysis of the senescent microenvironment in bone metastasis
Source: Cell Prolif. 2024 Sep 4;58(1):e13743. doi: 10.1111/cpr.13743 (PMC11693537; doi:10.1111/cpr.13743)
Supplement: Supplementary file 1 — Data S1. Supplementary figures. [file CPR-58-e13743-s010.docx]

**Supplementary information**

**Single-cell transcriptomic analysis of the senescent microenvironment in bone metastasis**

Shenglin Wang, Lu Ao, Huangfeng Lin, Hongxiang Wei, Zhaoyang Wu, Shuting Lu, Fude Liang, Rongkai Shen, Huarong Zhang, Tongjie Miao, Xiaopei Shen, Jianhua Lin, Guangxian Zhong


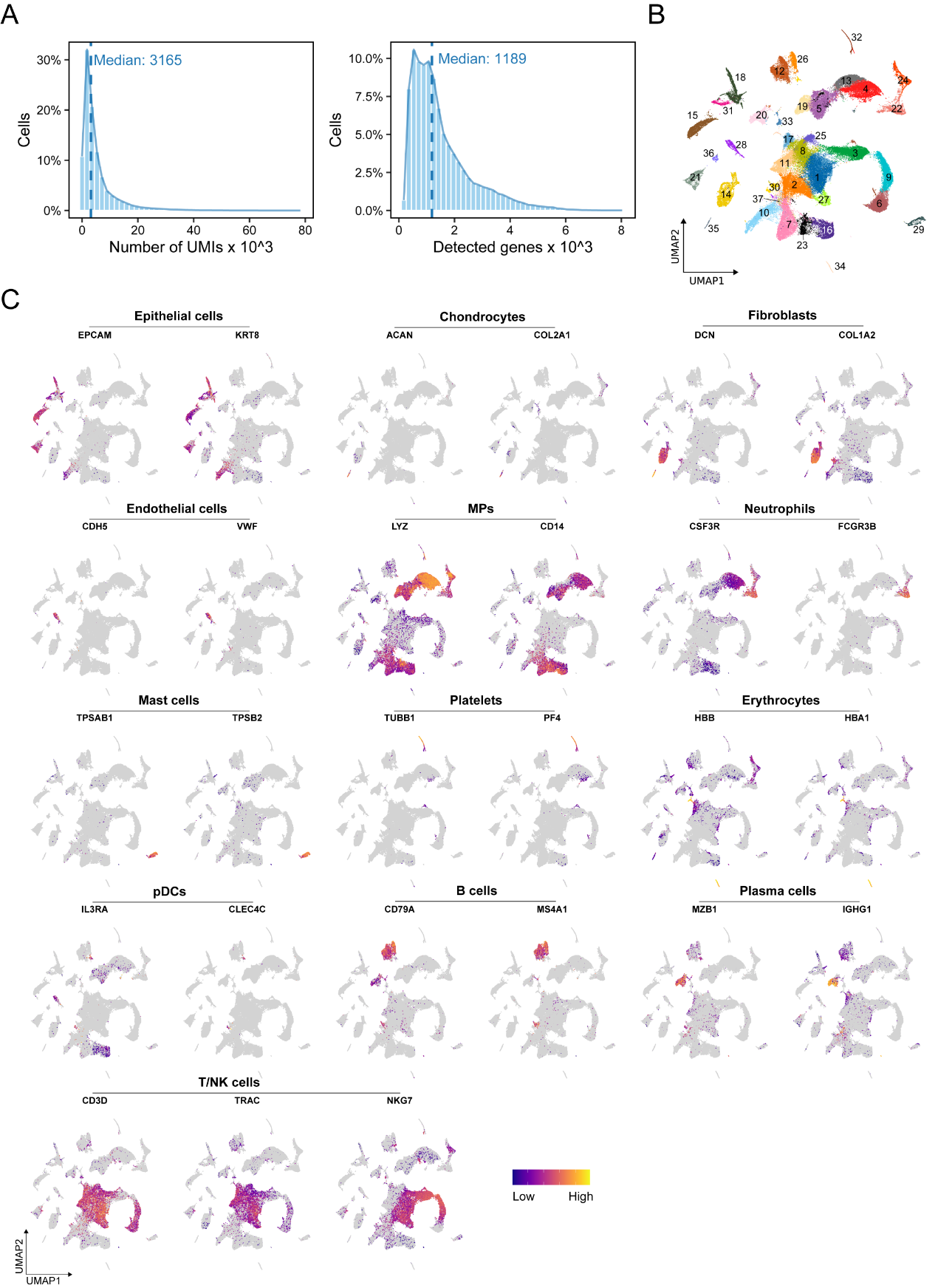


**FIGURE S1** The annotation of the NSCLC ecosystem. (A) Histogram indicating the number of unique molecular identifiers (UMIs) (left panel) and detected genes (right panel) for individual cells. (B) Uniform Manifold Approximation and Projection (UMAP) plot illustrating the annotated 37 clusters in the NSCLC ecosystem. (C) UMAP plot illustrating the expression of marker genes in the indicated cell types. NSCLC: non-small cell lung cancer.


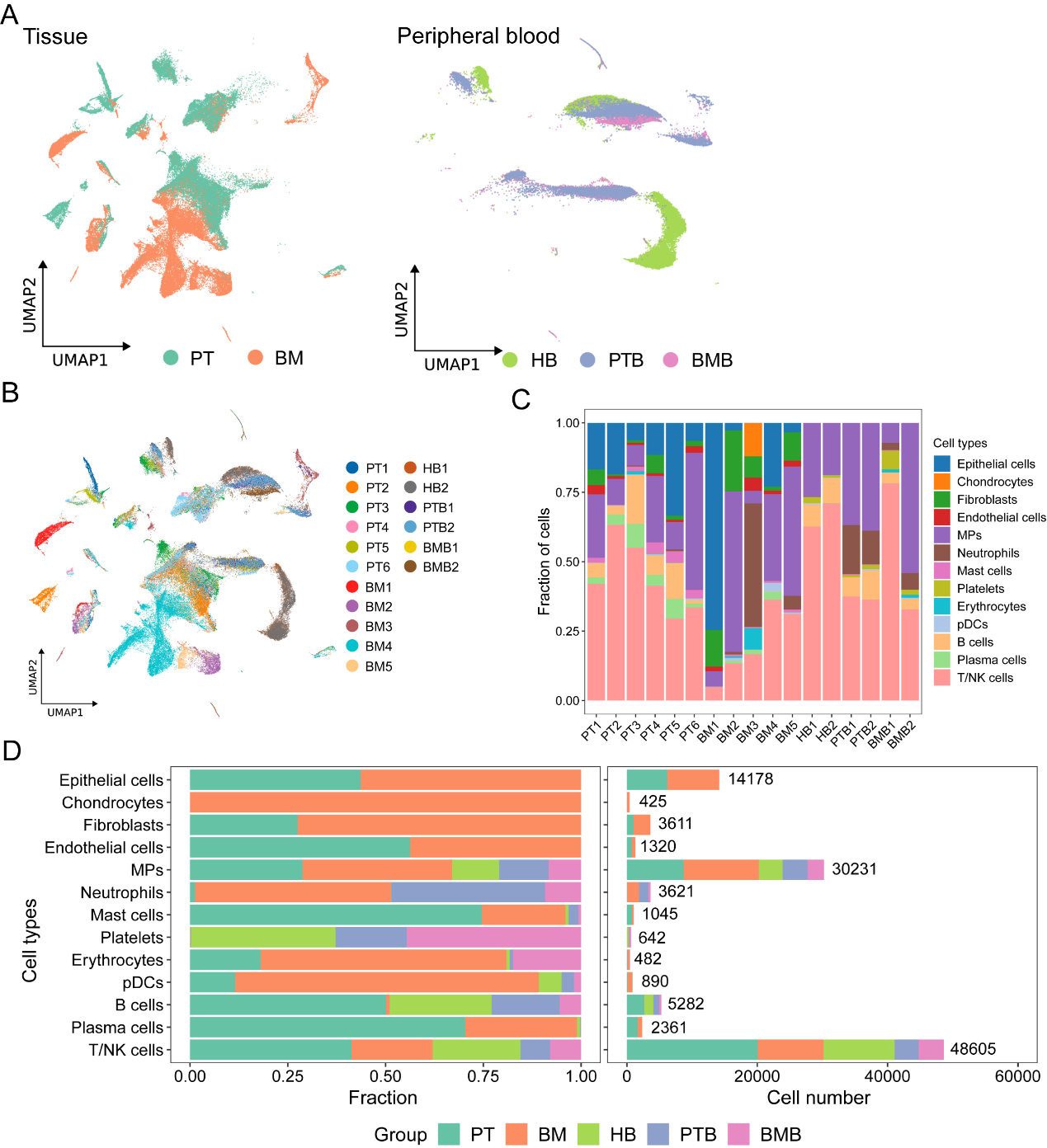


**FIGURE S2** The cell fraction of the NSCLC ecosystem. (A) Uniform Manifold Approximation and Projection (UMAP) plots depicting cells from tissues and peripheral blood colored by their respective groups. (B) UMAP plot depicting cells from tissues and peripheral blood colored based on samples. (C) Bar plot depicting the fraction of cell types for each patient. (D) Bar plot displaying the cell fraction (left panel) and cell number (right panel) from different groups for each cell type. NSCLC: non-small cell lung cancer.

**
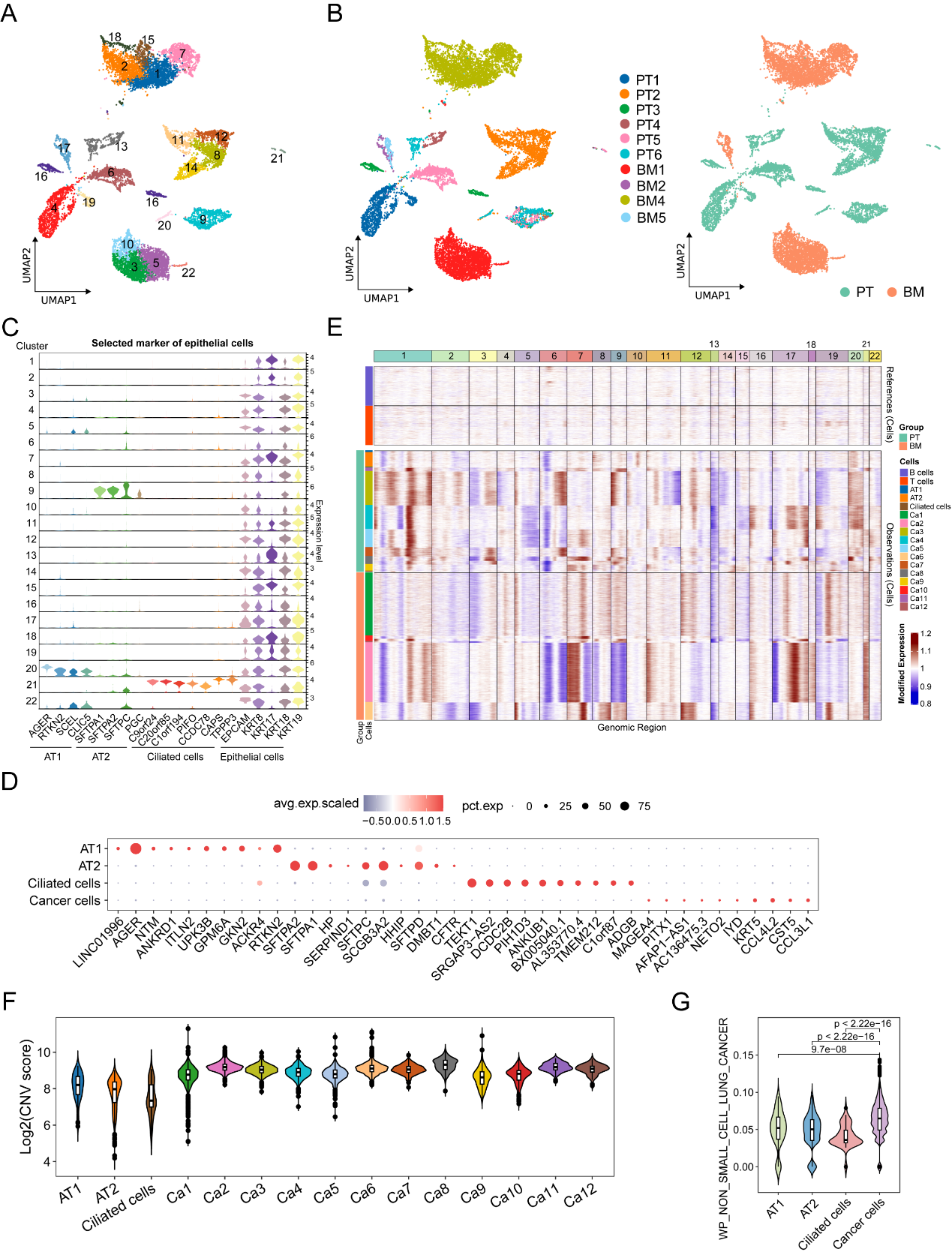
**

**FIGURE S3** Characteristics of epithelial cells in primary tumors (PT) and bone metastases (BM). (A) Uniform Manifold Approximation and Projection (UMAP) plot illustrating the annotated 22 clusters of epithelial cells. (B) UMAP plots illustrating epithelial cells colored based on samples (left panel) and groups (right panel). (C) Violin plot illustrating the expression of selected markers in the annotated 22 clusters. (D) Dot plot illustrating the expression of the top ten markers in each annotated cell type. (E) Heatmap illustrating the predicted CNV level in annotated cell clusters. (F) Violin-boxplot displaying the CNV score in annotated cell clusters. (G) Violin-boxplot displaying the interested signature score in epithelial cell types.


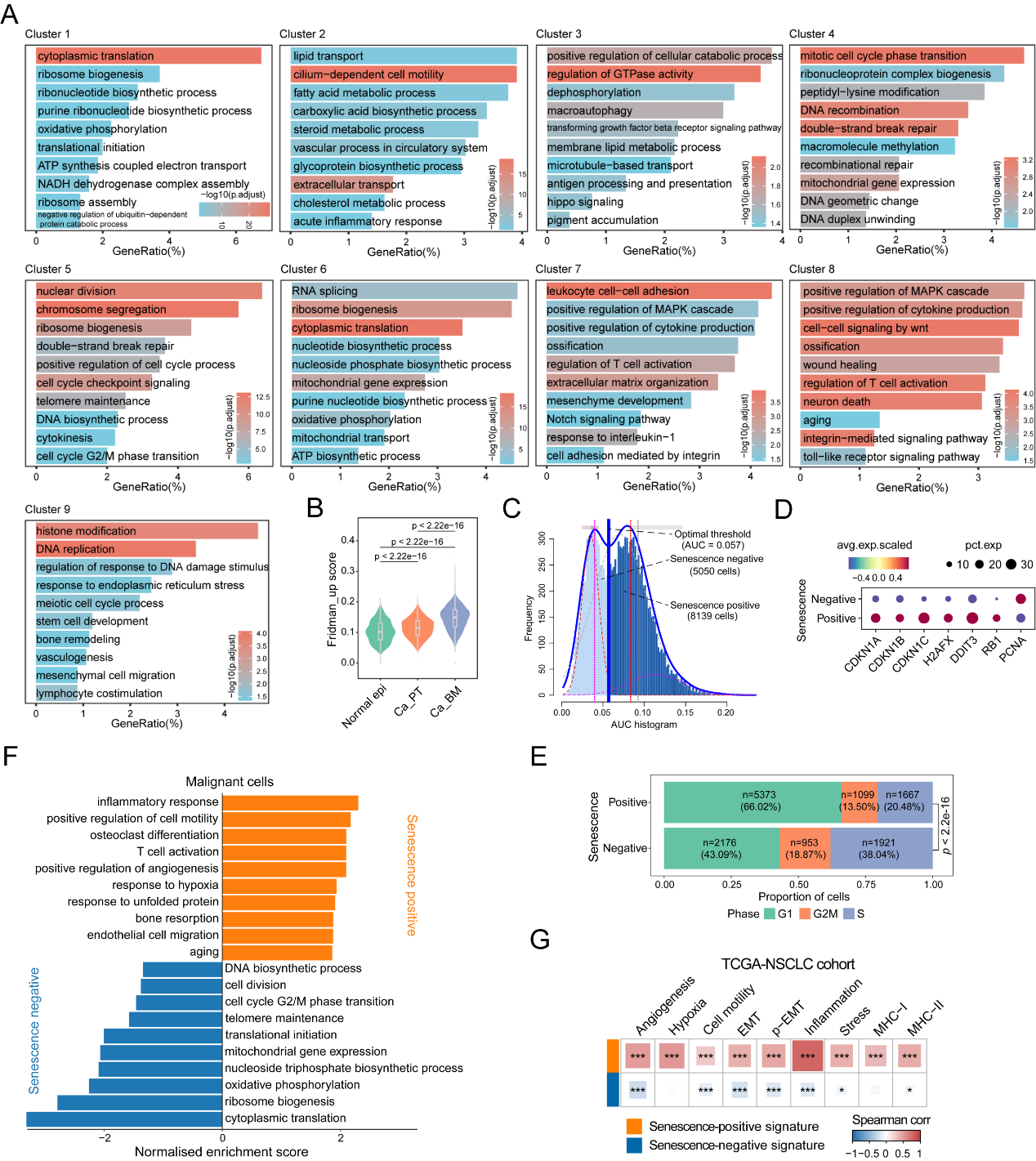


**FIGURE S4** Senescent characteristics of epithelial cells in primary tumors (PT) and bone metastases (BM). (A) The enriched biological process terms based on the cluster-specific genes from the mFuzz analysis. (B) Violin-boxplot displaying Fridman_up score of normal epithelial cells, Ca_PT and Ca_BM. (C) AUC score of the SenMayo signature with an optimal threshold value to determine senescent cells. (D) Dot plot displaying the expression of selected genes in senescence-positive and -negative cells. (E) The proportion of cell cycle phases in senescence-positive and -negative cells. *P* value was calculated by the Chi-square test. (F) Gene Set Enrichment Analysis of biological process terms based on the differentially expressed genes between senescence-positive and -negative malignant cells. (G) Heatmap illustrating the Spearman correlation of the senescence-positive and -negative signatures with interested signatures in the TCGA-NSCLC cohort. *** *P*-value < 0.001, * *P*-value < 0.05. NSCLC: non-small cell lung cancer.


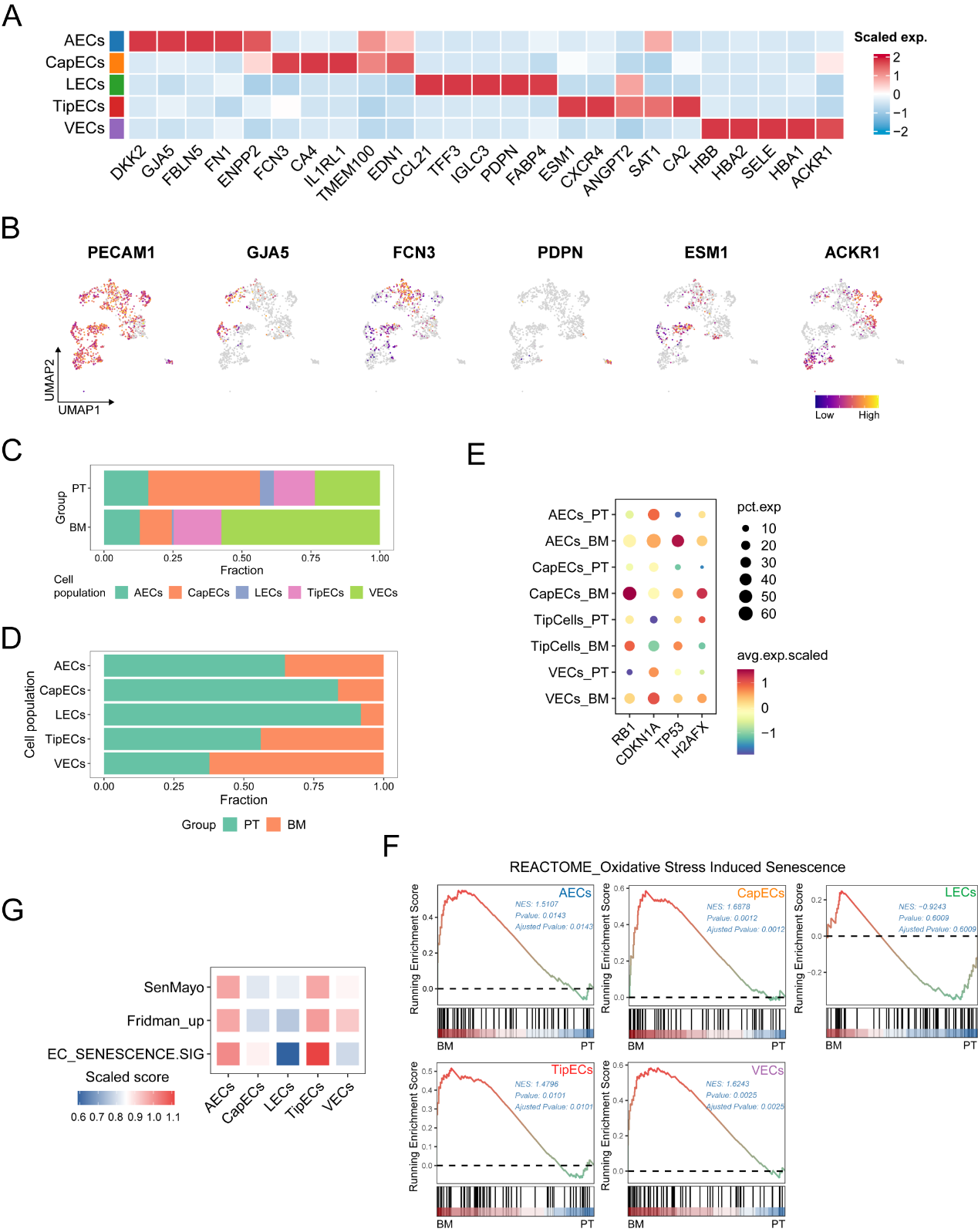


**FIGURE S5** Single-cell profiles of endothelial cells in primary tumors (PT) and bone metastases (BM). (A) Heatmap showing the expression of top five markers in annotated cell types. (B) Uniform Manifold Approximation and Projection (UMAP) plot showing the expression of selected markers in subclustered endothelial cells. (C) Bar plot displaying the proportion of cell subtypes for each group. (D) Bar plot displaying the proportion of cells from different groups for each cell subtype. (E) Dot plot displaying the expression of selected genes in VasECs from PT and BM. (F) Gene Set Enrichment Analysis of the Reactome pathway based on the differentially expressed genes across endothelial cell subtypes between BM and PT. (G) Heatmap showing the scores of the senescent signatures in endothelial cell subtypes.

**
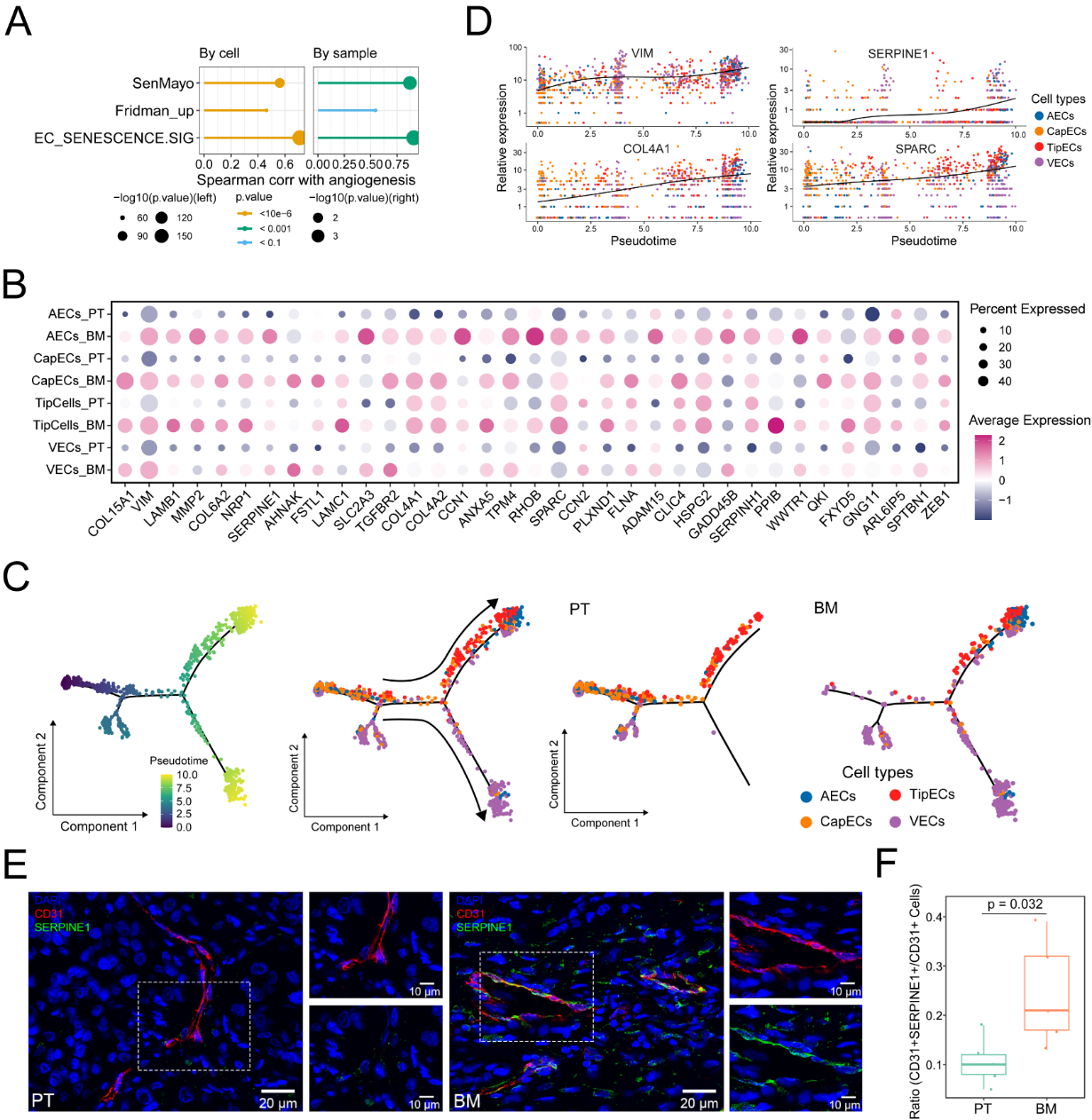
**

**FIGURE S6** Genes associated with senescence in endothelial cells. (A) Lollipop plots illustrating the Spearman correlation of the angiogenesis signature with the senescent signatures by cells (left panel) and by samples (right panel) in VasECs. (B) Dot plot displaying the scaled expression of mesenchymal markers in VasECs from different groups. (C) The evolutionary trajectory of VasECs colored by pseudotime (left first panel), by cell clusters (left second panel) and split by origin (right two panels). (D) The expression of mesenchymal markers in the pseudotime-ordered VasECs. (E-F) Representative immunofluorescence images (E) and boxplot (F) showing SERPINE1 expression level in PT and BM. Scale bar: 20 μm (200×) and 10 μm (400×). PT, primary tumor; BM, bone metastases.


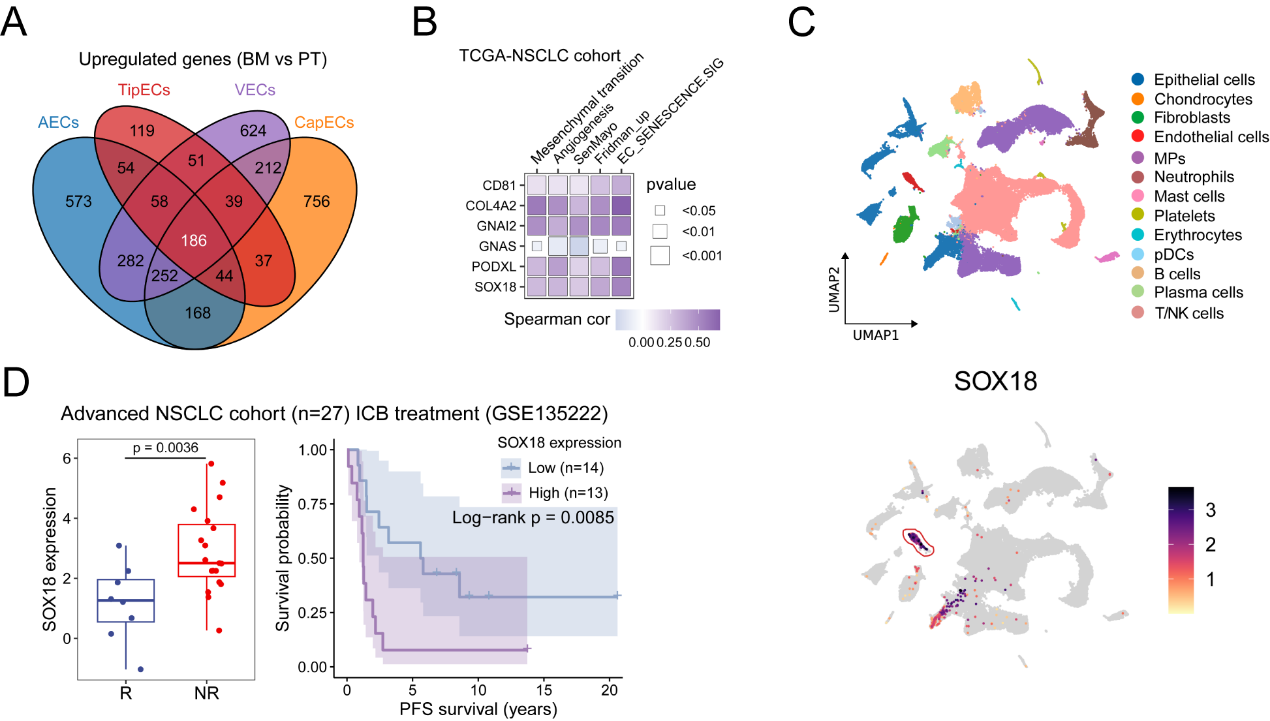
**FIGURE S7** SOX18 activation is associated with endothelial senescence in the bone-metastatic microenvironment. (A) Venn diagram showing the intersection of the upregulated genes in BM compared to PT across the VasECs subtypes. (B) Heatmap illustrating the Spearman correlation of selected genes with interested signatures in the TCGA-NSCLC cohort. (C) UMAP plot illustrating the annotated 13 cell types in the integrated cell map (upper panel) and SOX18 expression (lower panel) in the 13 annotated cell types. (D) The SOX18 expression in responders (R) and non-responders (NR) with ICB treatment (left panel) and the difference in progression-free survival (PFS) probability (%) post-treatment (right panel) according to SOX18 expression with the optimal cutoff in an independent NSCLC cohort. PT, primary tumor; BM, bone metastases; ICB, immune checkpoint blockade; NSCLC: non-small cell lung cancer.


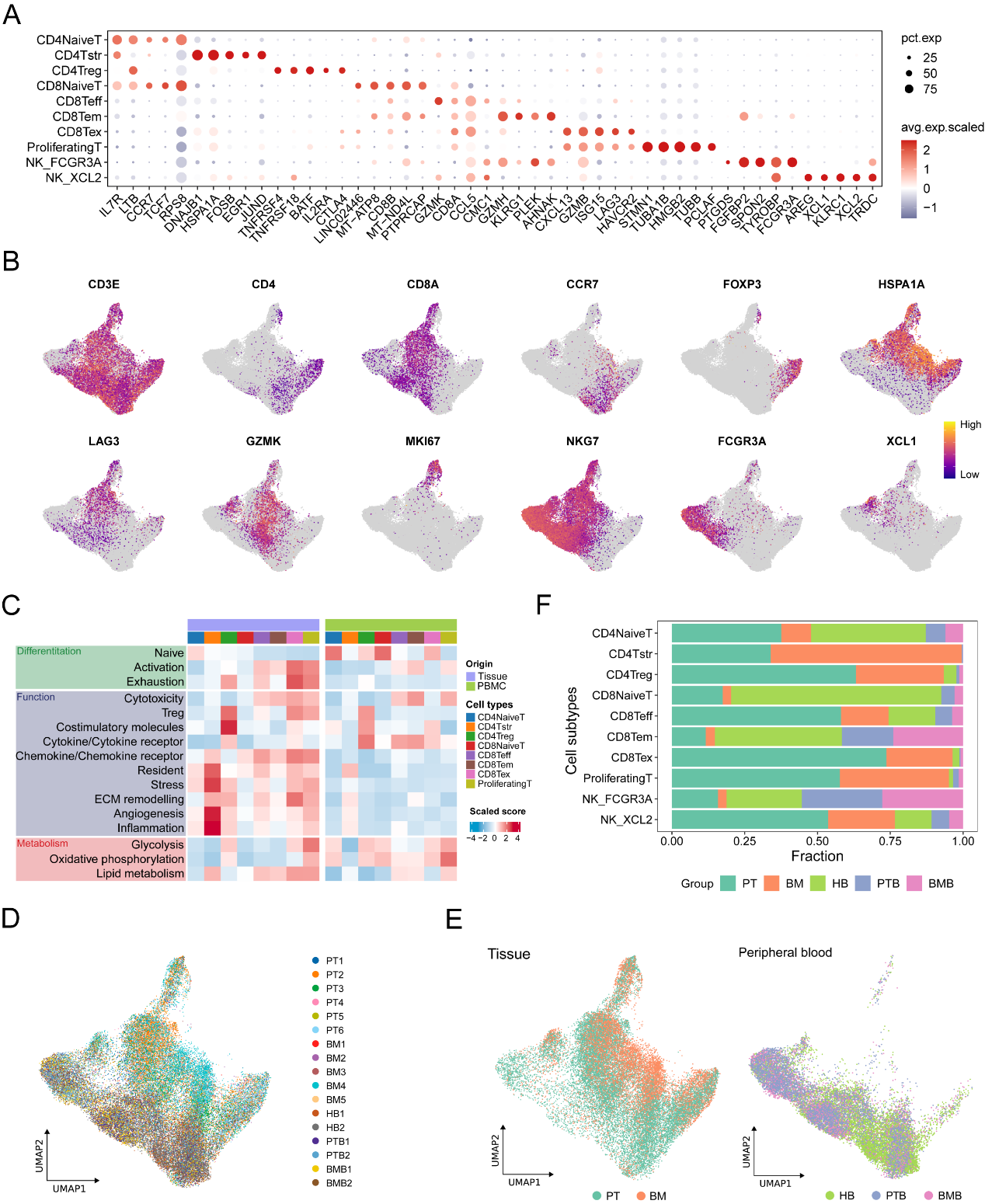


**FIGURE S8** Single-cell profiles of T/NK cells. (A) Dotplot showing the expression of top five markers in annotated cell types. (B) Uniform Manifold Approximation and Projection (UMAP) plot showing the expression of selected markers in subclustered T/NK cells. (C) Heatmap illustrating the scaled expression of 16 curated gene signatures across T-cell clusters from tissues and peripheral blood. (D) UMAP plot showing cells from tissues and peripheral blood colored based on samples. (E) UMAP plots showing cells from tissues and peripheral blood colored by their respective groups. (F) Bar plot displaying the proportion of cells from different groups for each cell subtype. NK: natural killer; PT, primary tumor; BM, bone metastases; HB: peripheral blood samples from healthy donors; PTB: peripheral blood samples from PT; BMB: peripheral blood samples from BM.

**
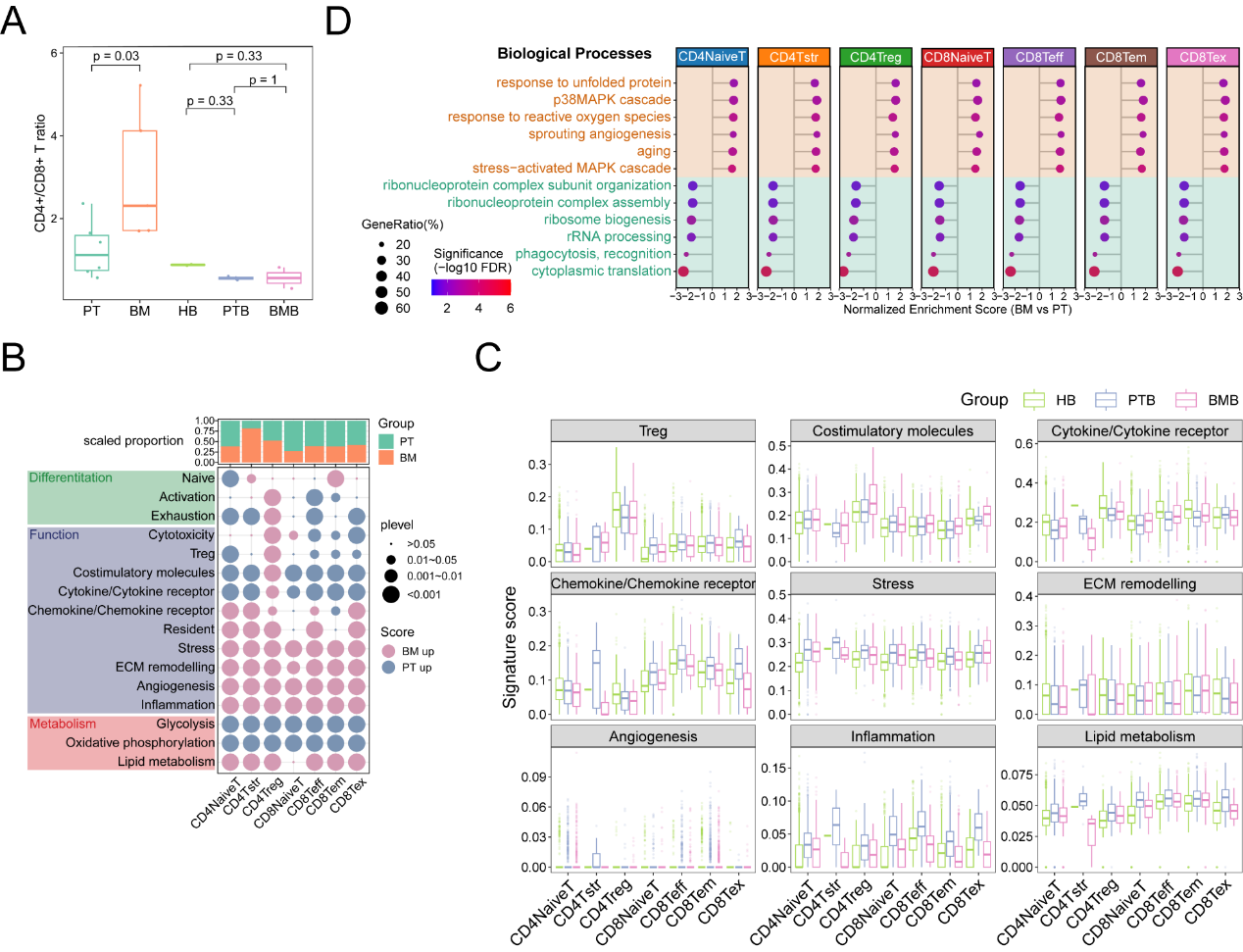
**

**FIGURE S9** Transcriptional heterogeneity of T cells between primary tumors (PT) and bone metastases (BM). (A) Boxplot indicating the CD4+/CD8+ T ratio in groups. (B) Dot plot (lower panel) illustrating the differential score of 16 signatures between PT and BM across T-cell subclusters, and bar plot (upper panel) displaying the scaled proportion of each subcluster in PT and BM. (C) Boxplot indicating the scores of 9 curated gene signatures in T-cell clusters across groups from peripheral blood. (D) Gene Set Enrichment Analysis of selected biological process terms based on the differentially expressed genes between PT and BM across the T-cell subclusters. HB: peripheral blood samples from healthy donors; PTB: peripheral blood samples from PT; BMB: peripheral blood samples from BM.


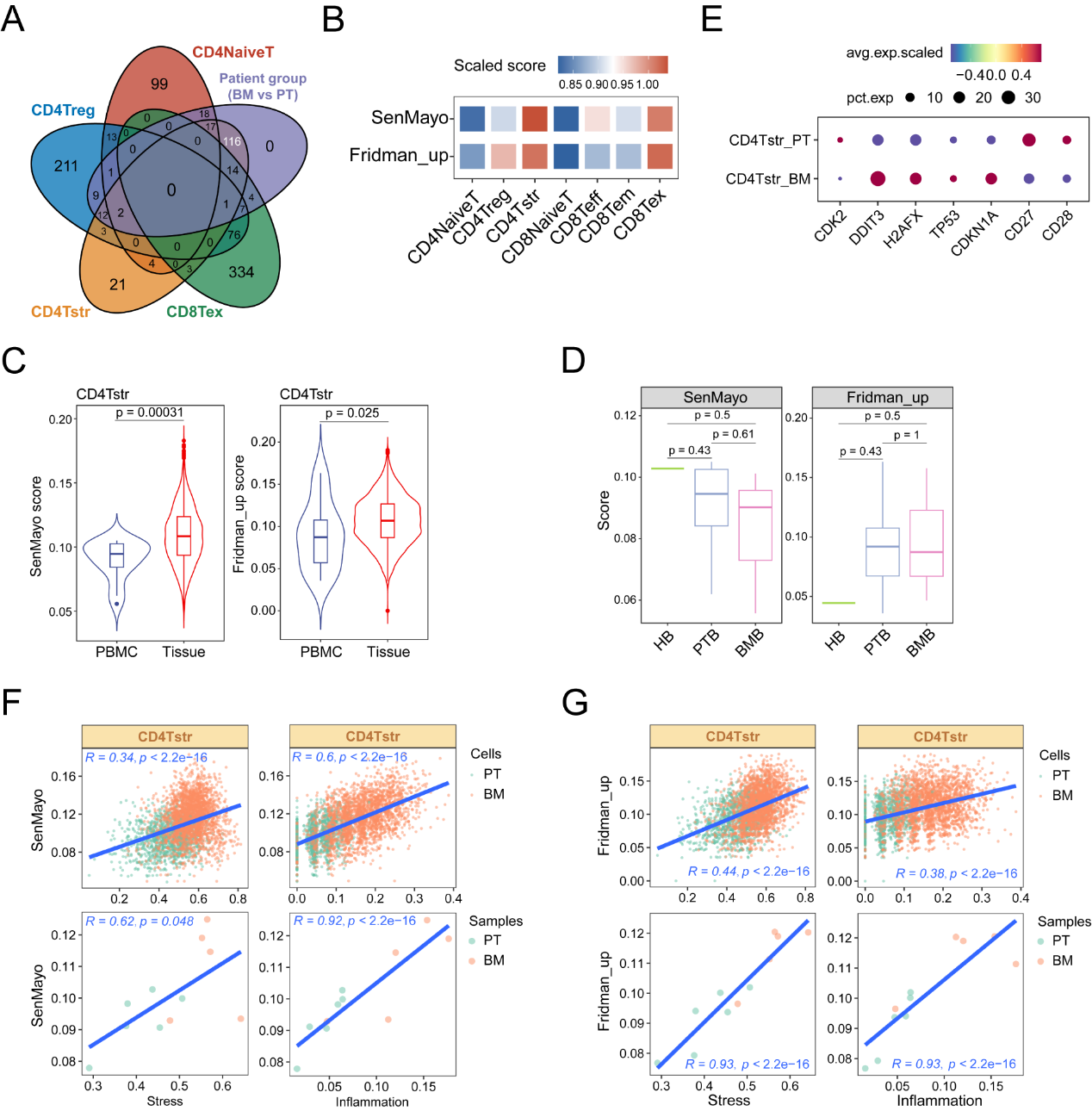


**FIGURE S10** Senescent characteristics of CD4Tstr cells in BM. (A) Venn diagram showing the intersection of differentially expressed genes (DEGs) between subclusters and between sample groups of T cells. (B) Heatmap illustrating the scaled scores of the senescence signatures across T-cell clusters from tissues and peripheral blood. (C) Violin-boxplots displaying the scores of the senescent signatures in the CD4Tstr cells from peripheral blood and tissues. (D) Boxplot indicating the scores of senescent signatures in CD4Tstr cells across HB, PTB, and BMB. (E) Dot plot displaying the expression of selected genes in CD4Tstrs from PT and BM. (F) Spearman correlation of the SenMayo signature with interested signatures by cells (upper panel) and by samples (lower panel) in CD4Tstr cells. (G) Spearman correlation of the Fridman_up signature with interested signatures in CD4Tstr cells by cells (upper panel) and by samples (lower panel). PT: primary tumor; BM: bone metastases; HB: peripheral blood samples from healthy donors; PTB: peripheral blood samples from PT; BMB: peripheral blood samples from BM. PBMC: peripheral blood mononuclear cell.


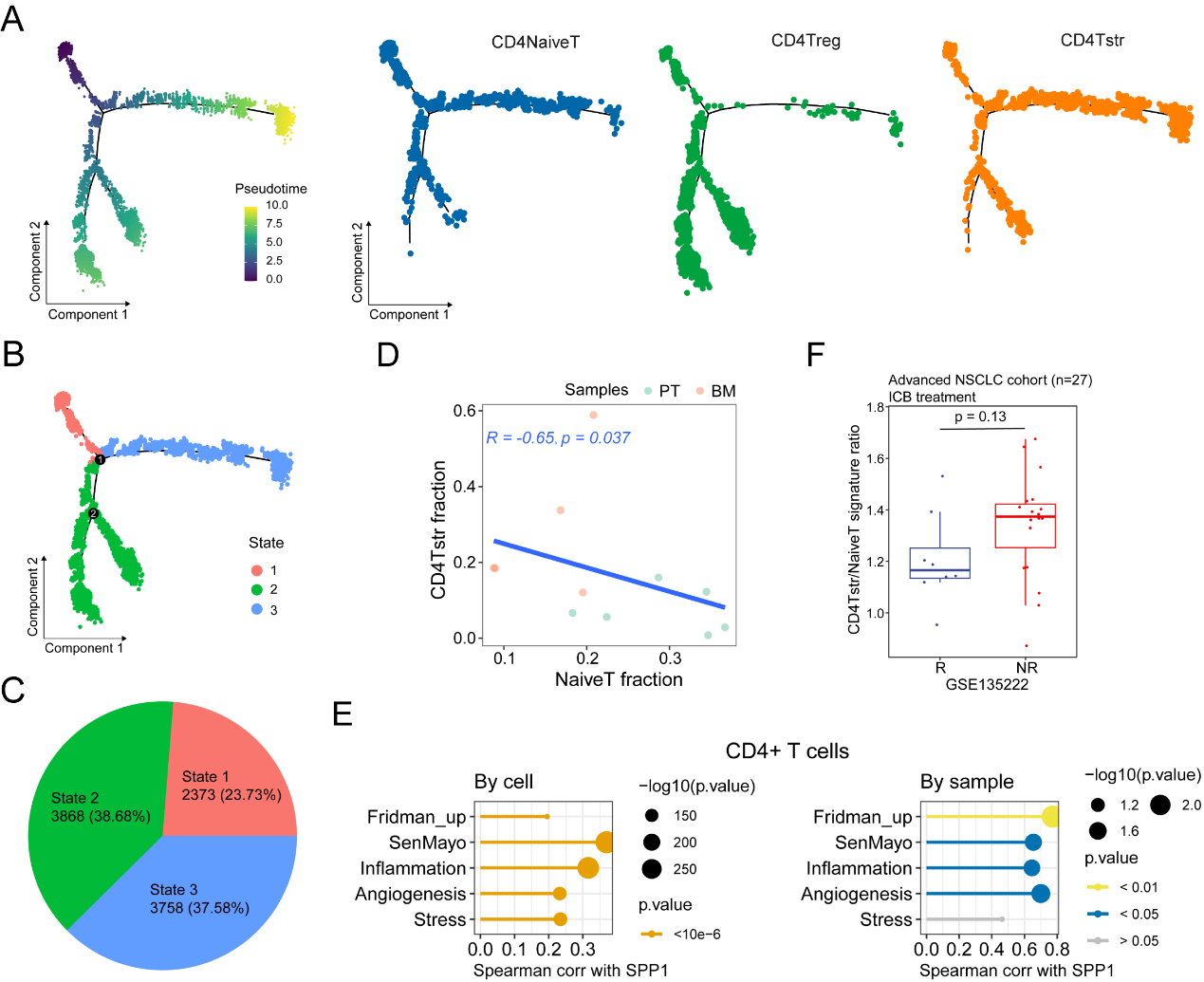


**FIGURE S11** Transition states of CD4+ T cells. (A) The evolutionary trajectory of CD4+ T cells, colored by pseudotime (left first panel) and split by cell clusters (right three panels). (B) The evolutionary trajectory of CD4+ T cells colored by cell states. (C) Pie chart showing the proportion of cells in each state as indicated in (B). (D) Spearman correlation of NaiveT fraction with CD4Tstr fraction for samples of tissue origin. (E) Lollipop plot illustrating the Spearman correlation of SPP1 with interested signatures by cells (left panel) and by samples (right panel) in CD4+ T cells. (F) Boxplot displaying the CD4Tstr/NaiveT signature ratio in responders (R) and non-responders (NR) patients in an independent NSCLC cohort with ICB treatment. ICB: immune checkpoint blockade. NSCLC: non-small cell lung cancer. PT: primary tumor; BM: bone metastases.

**
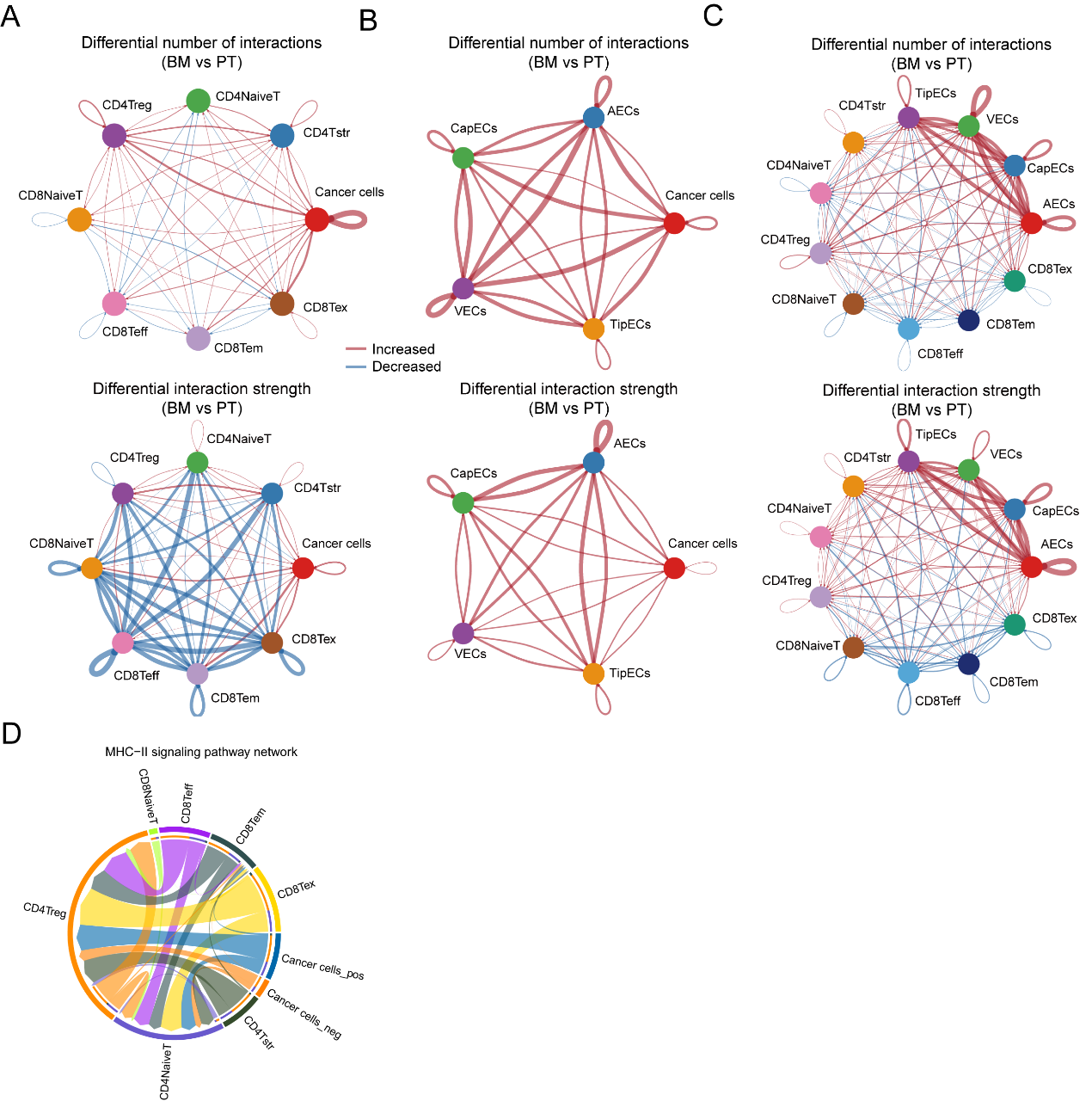
**

**FIGURE S12** Analysis of cell-cell communication networks in primary tumors (PT) and bone metastases (BM). (A) Comparison of the differential interaction number and strength of T cells and cancer cells between PT and BM. (B) Comparison of the differential interaction number and strength of VasECs and cancer cells between PT and BM. (C) Comparison of the differential interaction number and strength of T cells and VasECs between PT and BM. In (A), (B), and (C), the red line indicates the increased interaction number or strength in BM, and the blue line indicates decreased interaction number or strength in BM. (D) Chord plot presenting inferred intercellular communication network of MHC-II signaling pathway between senescence-positive (cancer cells_pos) and -negative (cancer cells_neg) cancer cells and T cells.

**
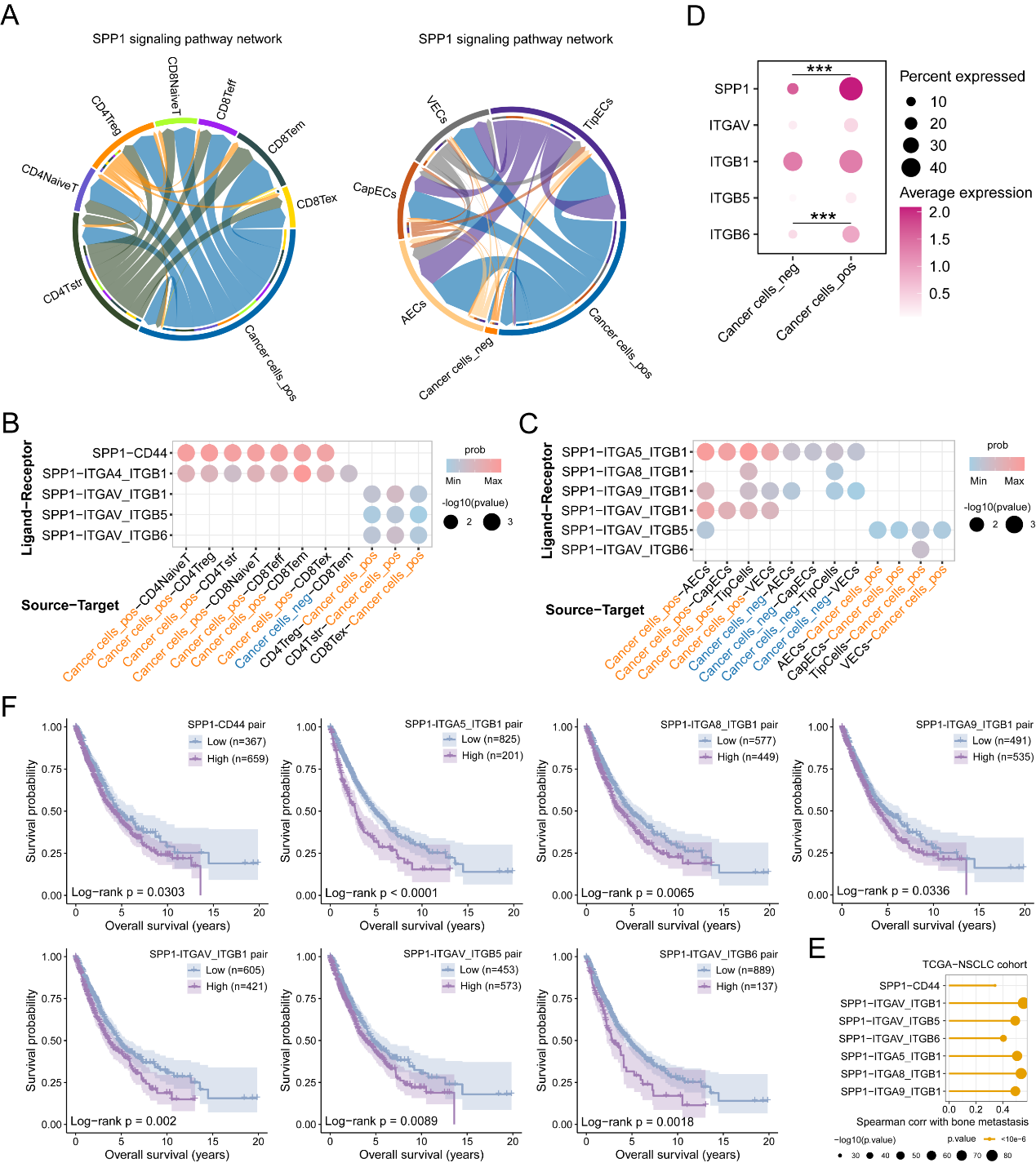
**

**FIGURE S13** Interaction activity of selected ligand-receptor pairs in SPP1 signaling pathway. (A) Chord plot presenting inferred intercellular communication network of SPP1 signaling pathway between senescence-positive (cancer cells_pos) and -negative (cancer cells_neg) cancer cells, T cells, and VasECs. (B-C) Dot plots showing significant ligand-receptor pairs that contribute to SPP1 signaling between senescence-positive (cancer cells_pos) and -negative (cancer cells_neg) cancer cells and T cells (B), between senescence-positive (cancer cells_pos) and -negative (cancer cells_neg) cancer cells and VasECs (C). (D) Dot plot showing the expression of selected genes in senescence-positive (cancer cells_pos) and -negative (cancer cells_neg) cancer cells. (E) Lollipop plot illustrating the Spearman correlation of ligand-receptor pairs with NSCLC bone metastasis assessed by the “VICENT_METASTASIS_UP” signature from MSigDB. (F) Kaplan-Meier plots showing the difference in survival probability (%) in patients with high versus low scores of the ligand-receptor pair signatures according to the optimal cutoff in the TCGA-NSCLC cohort. NSCLC: non-small cell lung cancer.
